# The ClpX and ClpP2 Orthologs of Chlamydia trachomatis Perform Discrete and Essential Functions in Organism Growth and Development

**DOI:** 10.1128/mBio.02016-20

**Published:** 2020-09-01

**Authors:** Nicholas A. Wood, Amanda M. Blocker, Mohamed A. Seleem, Martin Conda-Sheridan, Derek J. Fisher, Scot P. Ouellette

**Affiliations:** a Department of Pathology and Microbiology, College of Medicine, University of Nebraska Medical Center, Omaha, Nebraska, USA; b School of Biological Sciences, Southern Illinois University Carbondale, Carbondale, Illinois, USA; c Department of Pharmaceutical Sciences, College of Pharmacy, University of Nebraska Medical Center, Omaha, Nebraska, USA; Institut Pasteur

**Keywords:** *Chlamydia*, differentiation, division, protein turnover, protein quality control, Clp protease, ClpX, ClpP, CRISPRi, developmental cycle

## Abstract

Chlamydia trachomatis is an obligate intracellular bacterium that undergoes a complex developmental cycle in which the bacterium differentiates between two functionally and morphologically distinct forms, the elementary body (EB) and reticulate body (RB), each of which expresses its own specialized repertoire of proteins. Both primary (EB to RB) and secondary (RB to EB) differentiations require protein turnover, and we hypothesize that proteases are critical for mediating differentiation. The Clp protease system is well conserved in bacteria and important for protein turnover. Minimally, the system relies on a serine protease subunit, ClpP, and an AAA+ ATPase, such as ClpX, that recognizes and unfolds substrates for ClpP degradation. In *Chlamydia*, ClpX is encoded within an operon 3′ to *clpP2*. We present evidence that the chlamydial ClpX and ClpP2 orthologs are essential to organism viability and development. We demonstrate here that chlamydial ClpX is a functional ATPase and forms the expected homohexamer *in vitro*. Overexpression of a ClpX mutant lacking ATPase activity had a limited impact on DNA replication or secondary differentiation but, nonetheless, reduced EB viability with observable defects in EB morphology noted. Conversely, overexpression of a catalytically inactive ClpP2 mutant significantly impacted developmental cycle progression by reducing the overall number of organisms. Blocking *clpP2X* transcription using CRISPR interference led to a decrease in bacterial growth, and this effect was complemented in *trans* by a plasmid copy of *clpP2*. Taken together, our data indicate that ClpX and the associated ClpP2 serve distinct functions in chlamydial developmental cycle progression and differentiation.

## Abstract (precis)

Chlamydia trachomatis is the leading cause of infectious blindness globally and the most reported bacterial sexually transmitted infection both domestically and internationally. Given the economic burden, the lack of an approved vaccine, and the use of broad-spectrum antibiotics for treatment of infections, an understanding of chlamydial growth and development is critical for the advancement of novel targeted antibiotics. The Clp proteins comprise an important and conserved protease system in bacteria. Our work highlights the importance of the chlamydial Clp proteins to this clinically important bacterium. Additionally, our study implicates the Clp system playing an integral role in chlamydial developmental cycle progression, which may help establish models of how *Chlamydia* spp. and other bacteria progress through their respective developmental cycles. Our work also contributes to a growing body of Clp-specific research that underscores the importance and versatility of this system throughout bacterial evolution and further validates Clp proteins as drug targets.

## INTRODUCTION

Chlamydia trachomatis is the leading cause of both bacterial sexually transmitted infections (STIs) and infectious blindness worldwide (1, 2). When left untreated in women, STIs can result in chronic sequelae, including pelvic inflammatory disease, ectopic pregnancy, and tubal infertility. A better understanding of C. trachomatis molecular processes may help reveal essential systems that can be leveraged for more targeted intervention strategies.

C. trachomatis is an obligate intracellular bacterial pathogen that differentiates between distinct functional and morphological forms during the course of its developmental cycle (see reference 3 for review). The elementary body (EB) is small (∼0.3 μm in diameter), infectious, but nondividing (4, 5). EBs attach to host cells and are internalized into host membrane-derived vacuoles that are rapidly modified into the inclusion (6–9). Within this inclusion, EBs undergo primary differentiation into larger (∼1.0 μm in diameter) reticulate bodies (RBs). RBs are noninfectious but divide using a polarized budding mechanism (10) until secondary differentiation from RBs to EBs occurs. Many studies have detailed the transcriptional and proteomic differences between EBs and RBs (11–14). Given that chlamydial differentiation is not preceded by an unequal division and redistribution of intracellular proteins, as occurs in other bacteria such as Bacillus subtilis (see reference 15 for review) or Caulobacter crescentus (16) and that EBs and RBs have distinct proteomes, we hypothesize that proteomic turnover plays an integral role in chlamydial differentiation.

Previously, our groups characterized the two ClpP paralogs of C. trachomatis. We established that the *clp* protease-associated genes are expressed mid-developmental cycle and that ClpP1 and ClpP2 perform unique functions in chlamydial physiology based on differential effects related to overexpression of catalytically inactive mutants (17). In addition to the two ClpP paralogs, C. trachomatis harbors ClpX and ClpC orthologs (18). ClpX and ClpC are type I AAA+ (ATPase associated with diverse cellular activities) unfoldases that utilize ATP hydrolysis to linearize protein substrates for either degradation by the ClpP protease or refolding (19, 20). Type I AAA+ ATPases encode Walker A and Walker B motifs, which are responsible for ATP binding and hydrolysis, respectively (21, 22). These AAA+ unfoldases oligomerize to form homohexamers that then recognize substrates through multiple different mechanisms (see reference 23 for review).

Here, we characterized the role of ClpX in chlamydial growth and development. Because ClpX is encoded within an operon with *clpP2*, we also investigated effects of overexpression and knockdown of both components. C. trachomatis ClpX is highly conserved, possesses ATPase activity, and forms the expected homohexamer *in vitro*. Interestingly, overexpression of wild-type ClpX, ClpP2, and ClpP2X constructs in C. trachomatis had little effect on bacterial growth, but overexpression of the inactive mutants (alone or in tandem) had a substantial negative effect on recoverable inclusion-forming units (IFUs). However, the reduction in IFUs upon inactive ClpX overexpression resulted from nonfunctional EB generation, while the IFU reduction upon inactive ClpP2 overexpression was the result of a block in developmental cycle progression. Our results indicate that chlamydial ClpX is a true ortholog of bacterial ClpX and that the ClpP2X gene products are integral to chlamydial growth and development.

## RESULTS

### The chlamydial ClpX retains conserved motifs of, and exhibits predicted structural homology to, ClpX orthologs.

**FIG 1:**
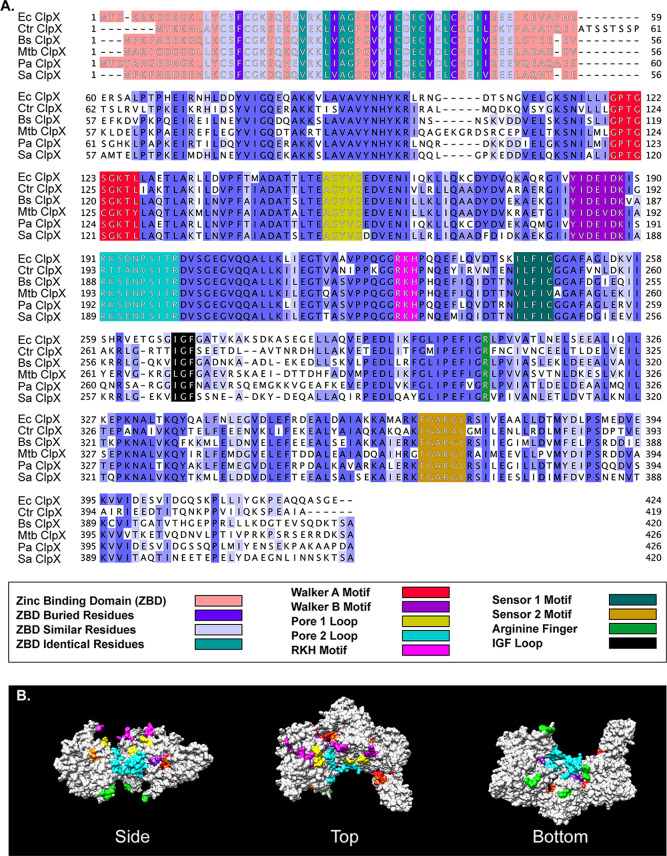
Bioinformatic analysis of chlamydial ClpX supports its role as an AAA+ ATPase. (A) Multiple-sequence alignment of chlamydial ClpX with the ClpX orthologs of various other bacteria. Ec, Escherichia coli; Ctr, Chlamydia trachomatis; Bs, Bacillus subtilis; Mtb, Mycobacterium tuberculosis; Pa, Pseudomonas aeruginosa; Sa, Staphylococcus aureus. Alignment was performed using Clustal Omega with default settings and presented using Jalview version 2. Alignment was colored by percent identity in shades of blue or as indicated below the alignment. (B) 3D model of ClpX was generated using SWISS-MODEL and presented in UCSF Chimera. Conserved motifs colored as for panel A except the IGF loops, which are colored lime green. Two subunits of the hexamer were hidden for easier visualization into the complex. Top and bottom are representations following a 90° rotation either clockwise or counterclockwise around the *x* axis. Of note, this model was generated using an ADP-bound form of ClpX as a template.

To initiate our study, we first performed bioinformatics and *ab initio* structural modeling analyses to determine whether the chlamydial ClpX (ClpX_Ctr_) possesses the expected conserved regions and motifs consistent with its proposed function as an AAA+ ATPase. Using multiple-sequence alignment, we aligned ClpX_Ctr_ to ClpX orthologs and annotated conserved motifs identified in other studies (Fig. 1A). ClpX_Ctr_ retains the N-terminal metal-binding domain (24, 25), the Walker A and B motifs for ATP binding and hydrolysis, respectively (21, 23), the sensor motifs for recognition of nucleotide bound state (26), the RKH motif and pore loops for substrate recognition (27–29) and unfolding (30, 31), the arginine finger for intersubunit sensing of nucleotide state in the ClpX hexamer (22, 32), and the IGF loop for interaction with ClpP (33, 34). Interestingly, the predicted secondary structure of ClpX_Ctr_ shows few notable aberrations (see Discussion) from other prototypical bacterial ClpX orthologs and is predicted to form the expected homohexamer by structural modeling (Fig. 1B, two subunits removed for clarity). The spatial conservation of AAA+ and ClpX-specific motifs (colored in Fig. 1B as in the multiple-sequence alignment) indicates that the chlamydial ClpX likely functions using a mechanism similar or identical to that of other ClpX orthologs. Taken together, these *in silico* studies suggest that ClpX_Ctr_ functions as a canonical AAA+ ATPase.

### Chlamydial ClpX forms the expected homohexamer and possesses ATPase activity.

**FIG 2:**
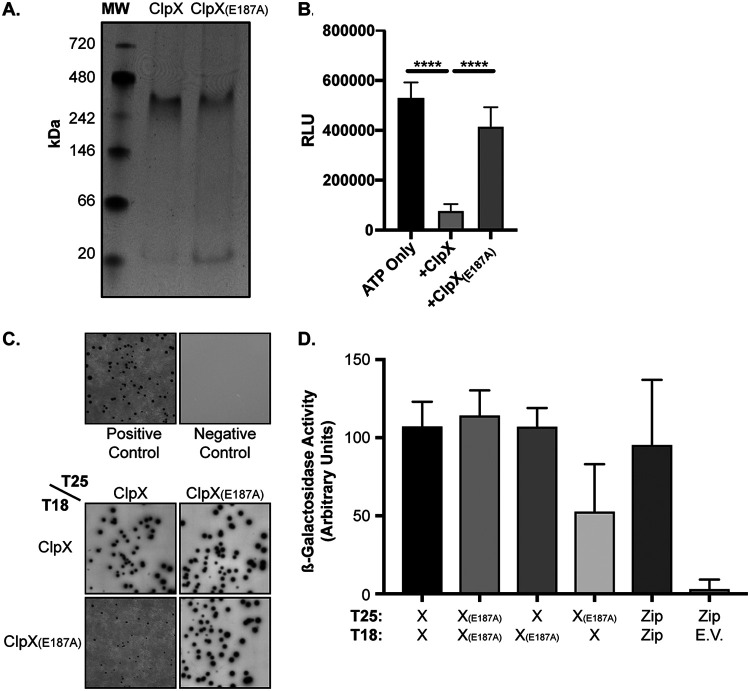
C. trachomatis ClpX is a functional ATPase that forms the expected hexamer. (A) Native PAGE assay of recombinant ClpX and ClpX_E187A_. Expected hexameric size is approximately 283 kDa. (B) ATP hydrolysis endpoint assay using Kinase-Glo. Relative luminescence units (RLU) are displayed on the *y* axis. Error bars represent standard deviations, and differences between samples are significant. ****, *P* < 0.0001, one-way analysis of variance (ANOVA). (C) Bacterial adenylate cyclase two-hybrid (BACTH) assays showing pairwise homotypic interaction of ClpX and ClpX_E187A_ as well as heterotypic interaction of ClpX and ClpX_E187A_. (D) β-Galactosidase activity of the BACTH interactions shown in panel C, displayed in arbitrary units on the *y* axis. All conditions *P* < 0.05 by unpaired *t* test compared to negative control.

To determine the oligomeric state of ClpX_Ctr_ *in vitro*, we purified recombinant protein and analyzed its migration by native PAGE. At the same time, we also constructed a Walker B ATPase mutant (E187A) ClpX_Ctr_ as a control for biochemical studies. Following the incubation of 10 μg of wild-type or mutant ClpX_Ctr_ for 20 min in a HEPES-based buffer, we loaded the entire volume into a 4% to 20% gradient gel. We observed the ClpX_Ctr_ proteins migrating above the 242-kDa band of the molecular weight ladder, which is close to the expected hexameric size of 283 kDa (Fig. 2A). We then sought to assess ATPase activity of recombinant wild-type and ATPase mutant ClpX_Ctr_ using the Kinase-Glo endpoint assay, which measures ATP remaining in a sample at the end of the reaction period. Indeed, ClpX_Ctr_ hydrolyzed ATP, while the ATPase mutant isoform showed a significant defect in ATP hydrolysis (Fig. 2B). These data indicate that ClpX_Ctr_ (i) forms a homohexamer of the predicted size and (ii) possesses ATPase activity that is abrogated by a mutation in the Walker B motif.

We next tested whether wild-type and ATPase mutant ClpX_Ctr_ interact with each other using the bacterial adenylate cyclase two-hybrid (BACTH) assay. This system is predicated on the reconstitution of adenylate cyclase activity by bringing two complementary fragments of the enzyme (T25 and T18) into close proximity by interacting proteins. Generation of cAMP by the reconstituted adenylate cyclase drives β-galactosidase production that can be measured qualitatively by the presence of blue colonies and growth on minimal medium (Fig. 2C) or quantitatively by measuring enzyme activity directly (Fig. 2D). We performed a series of pairwise interaction tests between the wild-type and mutant ClpX_Ctr_. In each instance, we observed a positive interaction that was quantifiable and on par with the positive control (T25-Zip versus T18-Zip). We conclude from these data that the mutant isoform can interact with the wild-type isoform.

### Overexpression of ATPase mutant ClpX or catalytically inactive ClpP2 has both distinct and overlapping effects.

**FIG 3:**
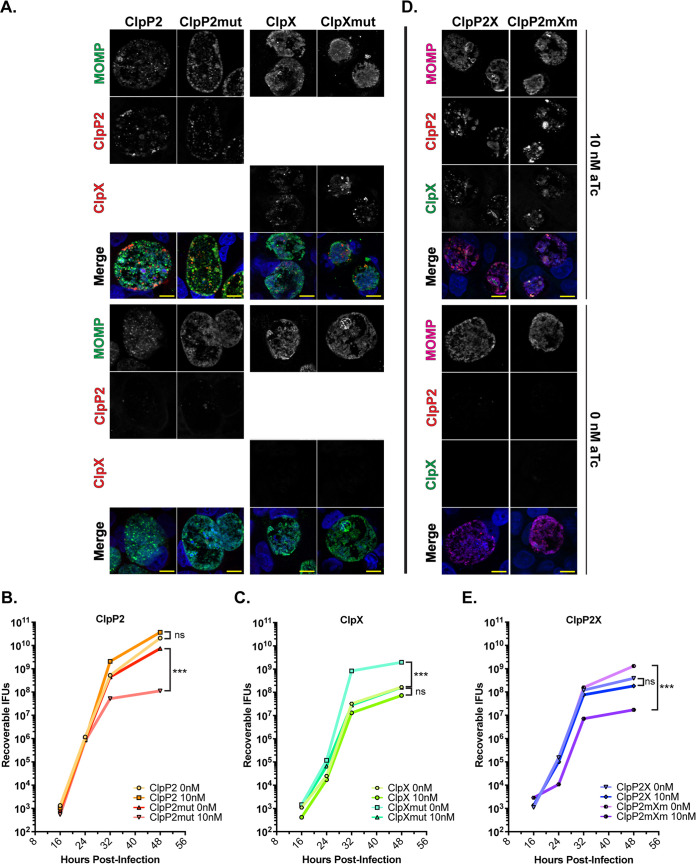
Overexpression of Clp mutant isoforms negatively impacts C. trachomatis at later time points. (A) IFA of ClpP2 and ClpX wild-type and mutant overexpression at 24 and 48 hpi. Samples were induced with 10 nM aTc at 10 hpi and stained for MOMP (green), 6×His (red), and DNA (blue). Bars, 10 μm. Images were acquired on a Zeiss Apotome at ×100 magnification. One-step growth curves of wild-type and mutant ClpP2 (B) or ClpX (C). Samples induced with 10 nM aTc at 10 hpi. IFUs recovered are displayed as log_10_ values. Values represent the averages from two independent experiments. (D) IFA of ClpP2X wild-type or mutant operons at 24 and 48 hpi. Samples stained for MOMP (pink), FLAG (ClpP2, red), 6×His (ClpX, green), and DNA (blue). Parameters as described for panel B. (E) One-step growth curves of ClpP2X wild-type and mutant overexpression. IFUs recovered are displayed as log_10_ values.

We previously measured the effects of overexpression of both wild-type and catalytically inactive ClpP2_Ctr_ on chlamydial growth and observed a modest reduction in growth at 24 h postinfection (hpi) (17). We wanted to more carefully assess growth differences during the chlamydial developmental cycle in the presence of overexpressed wild-type and mutant ClpX_Ctr_ and ClpX_E187A_, ClpP2_Ctr_ and ClpP2_S98A_, or both together (ClpP2X_Ctr_ and ClpP2_S98A_ClpX_E187A_). To do this, we assessed growth curves where we induced expression, or not, at 10 hpi and quantified growth at various time points after induction. Immunofluorescence analysis (IFA) of replicate treatments and quantification of recoverable inclusion forming units (IFUs; a proxy for EBs) revealed distinct effects upon overexpression of the individual components (Fig. 3A to C) as well as with the entire operon (Fig. 3D and E). We noted that overexpression of wild-type ClpP2_Ctr_ showed no appreciable effect at either 24 or 48 hpi (14- and 38-h pulses of induction, respectively), whereas overexpression of ClpP2_S98A_ appeared to reduce the number of organisms present within the inclusion at 48 hpi but not at 24 hpi (Fig. 3A). These observations correlated with a marked decrease in EB production in the later time points of mutant ClpP2_S98A_ but not wild-type ClpP2_Ctr_ overexpression (Fig. 3B). Conversely, ATPase mutant ClpX_E187A_ overexpression resulted in smaller inclusions and a decrease in IFUs (Fig. 3A and C). Effects on IFU recovery suggest that C. trachomatis is more sensitive to ClpX_Ctr_ than ClpP2_Ctr_ disruption earlier in the developmental cycle, as the IFU reduction is exacerbated sooner with ClpX_E187A_ overexpression (observe the differences at 24 hpi in Fig. 3B and C). As noted for the overexpression of individual wild-type isoforms, there was no significant impact on IFU recovery when overexpressing both wild-type ClpP2_Ctr_ and ClpX_Ctr_ in tandem. Consistent with the effects of overexpressing individual mutant isoforms, overexpression of the mutant ClpP2_S98A_ and ClpX_E187A_ isoforms in tandem showed an exacerbated phenotype throughout the developmental cycle, as noted by both IFA and IFU assays (Fig. 3D and E). Importantly, the wild-type chromosomal copies of ClpP2_Ctr_ and ClpX_Ctr_ continued to be expressed during these overexpression assays. Therefore, the true impact of overexpression of the mutant isoforms is likely underrepresented.

### Functional disruption of ClpP2 blocks developmental cycle progression while ClpX disruption reduces EB viability.

**FIG 4:**
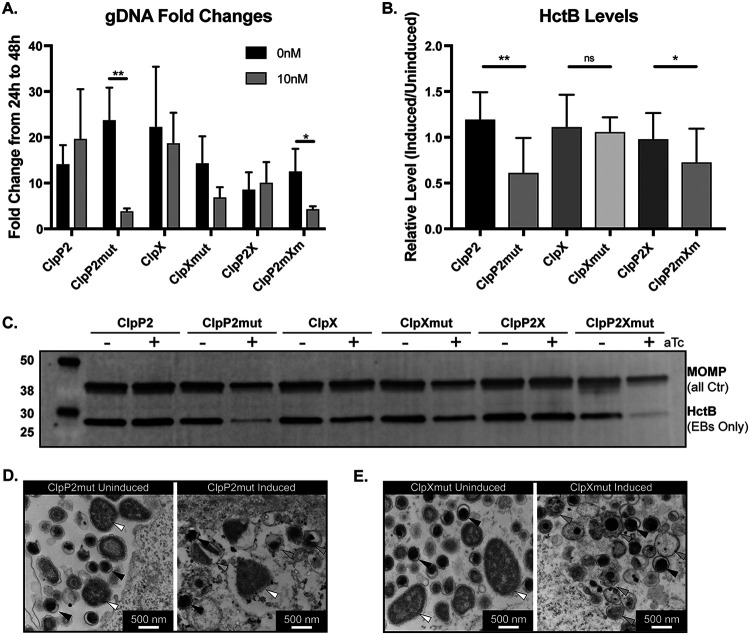
Functional disruption of ClpX and ClpP2 perturbs chlamydial development but in different manners. (A) Fold changes of detectable gDNA from 24 to 48 hpi for each strain. Samples were induced at 10 hpi with 10 nM aTc. *, *P* ≤ 0.05; **, *P* ≤ 0.001 by paired *t* test. Values are displayed as the averages from three independent experiments with error bars representing standard deviations. (B and C) Western blot analysis of HctB levels at 48 hpi with and without overexpression. One well of a six-well plate was lysed into 500 μl of denaturing lysis buffer; 50 μg of protein from the clarified lysate for each sample was assessed. Blots were probed for MOMP (IR680) and HctB (IR800). Grayscale blot shown is representative of three independent experiments. Quantified integrated density of the staining is shown in panel B. HctB levels were normalized to MOMP levels in each sample to account for differences in bacteria in each sample. Values displayed as levels of induced to uninduced HctB/MOMP ratios for each strain. *, *P* ≤ 0.05; **, *P* ≤ 0.001; ns, not significant by multiple-comparisons *t* test. (D and E) Representative images of electron micrographs of the indicated strains and conditions. White triangles indicate normal RBs, black triangles indicate normal EBs, and gray triangles indicate abnormal developmental forms.

Given that the IFU assay only measures EB viability from a population and not total bacterial numbers or differentiation status, we next wanted to address these nuances of the chlamydial developmental cycle. We first measured genomic DNA as a proxy for total number of bacteria (i.e., both RBs and EBs). Overexpression of any wild-type protein had no significant impact on DNA accumulation. From 24 hpi to 48 hpi, we observed a significant drop in the rate of increase of genomic DNA (gDNA) levels when ClpP2_S98A_ was overexpressed alone or in the mutant operon configuration (Fig. 4A). Overexpression of ClpX_E187A_ showed a trend (2-fold) toward decreased DNA levels that was not statistically significant. This was surprising given the roughly 20-fold reduction in IFUs (Fig. 3), suggesting total bacterial numbers during ClpX_E187A_ overexpression are unaffected.

To determine if there was an impact on differentiation status during overexpression of wild-type and mutant isoforms, we next assessed levels of HctB, an EB-specific gene product (35–37), by Western blotting as an indicator of secondary differentiation. We normalized the integrated density of HctB to the integrated density of major outer membrane protein (MOMP; present in both EBs and RBs) to ensure that we were comparing HctB levels to the total number of bacteria to give a more robust readout of differentiated bacteria within the population. The relative HctB levels in samples where ClpP2_S98A_ was overexpressed were significantly reduced as expected, indicating the generation of fewer EBs and consistent with IFU and genomic DNA data, whereas the other experimental conditions showed no changes in relative HctB levels (Fig. 4B and C). These data suggest that overexpression of ClpX_E187A_ does not impact total number of bacteria, as measured by gDNA levels, or RB-to-EB differentiation, as measured by HctB levels. Nevertheless, the recoverable IFUs were decreased when ClpX_E187A_ was overexpressed (Fig. 3C and E), indicating a defect in the EBs being produced.

To further explore this possibility, we prepared samples for transmission electron microscopy to examine at higher resolution the morphology of EBs and RBs from ClpP2_S98A_- and ClpX_E187A_-overexpressing strains. As RBs condense to EBs, an intermediate form can be observed where the chlamydial nucleic acids are visible as an electron dense spot. Consistent with other measured effects (Fig. 4D and E; see also Fig. S1A in the supplemental material), ClpP2_S98A_ overexpression resulted in smaller inclusions with fewer organisms, whereas ClpX_E187A_ appeared to have no effect on bacterial numbers (Fig. 4D and E). In both induced and uninduced samples for each strain, we noted normal RBs (Fig. 4D and E, white triangles) and normal EBs (Fig. 4D and E, black triangles). However, we observed numerous abnormal developmental forms following ClpX_E187A_ overexpression that appeared to be multinucleated or abnormally condensing intermediate forms (Fig. 4E, gray triangles). These data are consistent with the limited effect of ClpX_E187A_ overexpression on the production of HctB (Fig. 4B and C) but the decreased recoverable IFUs (Fig. 3A and C), further supporting that disruption of normal ClpX function may affect EB viability.

10.1128/mBio.02016-20.1 FIG S1 Transmission electron microscopy of the effects of overexpression of mutant Clp isoforms on chlamydial morphology. (A) Representative ClpP2_S98A_ uninduced or induced samples at 48 hpi. Samples were induced or not with 10 nM aTc at 10 hpi and were fixed and processed at 48 hpi. Arrows indicate abnormal forms in the induced samples. (B) Representative ClpX_E187A_ uninduced or induced samples at 48 hpi. Samples were treated, fixed, and processed as previously discussed. Arrows indicate abnormal forms with intrabacterial aggregates. (C and D) Enlargements of areas boxed in panel B. Download FIG S1, PDF file, 0.8 MB. Copyright © 2020 Wood et al. 2020 Wood et al. This content is distributed under the terms of the Creative Commons Attribution 4.0 International license.

### Knockdown of the *clpP2X* operon reduces recoverable progeny and results in reduced plasmid retention, while complementation with *clpP2* partially restores infectivity.

**FIG 5:**
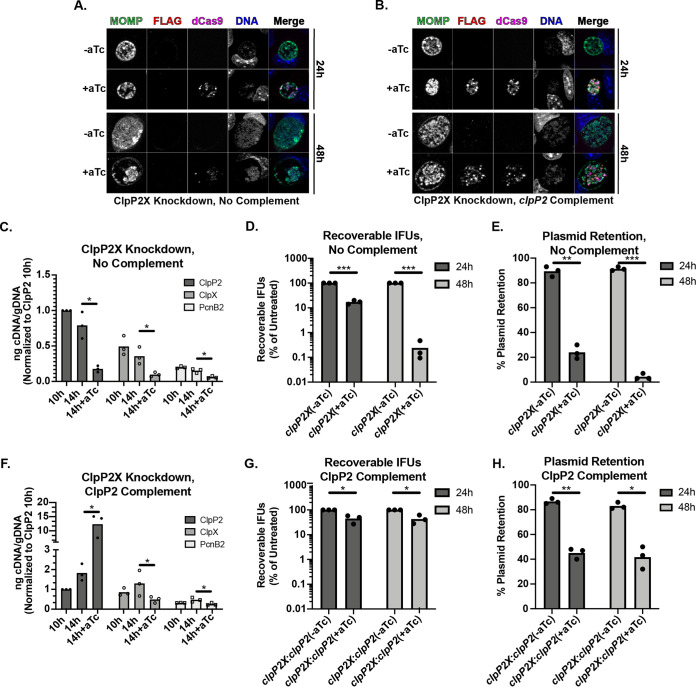
Knockdown of ClpP2X negatively impacts *Chlamydia*, while complementation with ClpP2 partially restores normal development. Representative immunofluorescence images of *clpP2X* knockdown with either (A) no complementation or (B) *clpP2* complementation. (C) Transcript levels upon knockdown of *clpP2X*. Data displayed are the values from three independent biological replicates of triplicate reverse transcription-qPCRs (RT-qPCRs). Values are normalized to the 10-h uninduced ClpP2 value for each experiment. Samples were induced using 10 nM aTc. (D) IFU titers following induction of knockdown at 4 hpi. Values are presented on a log_10_ scale percentage of the respective uninduced titer. (E) Plasmid retention based on the ratio of GFP-positive inclusions to the total number of inclusions is displayed as a percent for each condition. (F to H) IFU titers and plasmid retention of *clpP2X* knockdown with *clpP2* complementation. Conditions used are the same as for panels C to E.

Overexpression of mutant isoforms of ClpP2_Ctr_ and/or ClpX_Ctr_ was sufficient to disrupt chlamydial development in the presence of endogenous ClpP2X_Ctr_. However, we wanted to directly block the chromosomal copies by employing an improved version of the chlamydial CRISPR interference (CRISPRi) strategy previously described by us (38 and data not shown). CRISPRi relies on the inducible expression of a catalytically inactive Cas9 (dCas9) in combination with a guide RNA (gRNA) to block transcription at specific chromosomal sites (39). We transformed C. trachomatis L2 with vectors encoding inducible dCas9 and constitutive gRNAs targeting either the *clpP2X* or *incA* intergenic regions (Fig. 5A; see also Fig. S2 and S3). Of note, *pcnB2*, a predicted poly(A) polymerase, is located at the 3′ end of the *clpP2X* operon and was included in our analyses. To determine whether we could also complement the effects of knockdown, we constructed vectors harboring either *clpP2_FLAG* or *pcnB2_FLAG* in a transcriptional fusion 3′ to dCas9. These vectors were transformed into C. trachomatis L2. Under conditions when dCas9 was expressed, ClpP2_FLAG or PcnB2_FLAG was also expressed (Fig. 5B and Fig. S4). IncA knockdown served as a control, since *incA* is a nonessential gene (40). These CRISPRi transformants were used to infect HEp-2 cells.

10.1128/mBio.02016-20.2 FIG S2 (A) RT-qPCR of *clpP1*, *euo*, and *omcB* upon *clpP2X* knockdown. Data shown are the averages from three biological replicates, each with three technical replicates. Values are normalized to the 10-h timepoint for each respective gene. Error bars represent standard deviations. Samples induced as indicated with 10 nM aTc. (B) RT-qPCR of *clpP2X* knockdown with *pcnB2* complementation. Values normalized to the ClpP2 10-h samples. Download FIG S2, PDF file, 0.4 MB. Copyright © 2020 Wood et al. 2020 Wood et al. This content is distributed under the terms of the Creative Commons Attribution 4.0 International license.

10.1128/mBio.02016-20.3 FIG S3 (A) Immunofluorescence staining to confirm knockdown of IncA upon induction of dCas9 expression. Sa_dCas9 was induced or not at 4 hpi. Samples were harvested at 24 hpi and were stained for chlamydial MOMP (green), IncA (magenta), and DNA (Blue). Bars, 10 μm. (B and C) Recoverable IFUs and plasmid retention upon *incA* knockdown. Download FIG S3, PDF file, 2.8 MB. Copyright © 2020 Wood et al. 2020 Wood et al. This content is distributed under the terms of the Creative Commons Attribution 4.0 International license.

10.1128/mBio.02016-20.4 FIG S4 (A) Representative immunofluorescence images of *clpP2X* knockdown with *pcnB2* complementation. Samples were induced as in IFU experiments. Chlamydial MOMP in green, FLAG (complemented gene) in red, dCas9 in magenta, and DNA in blue. (B) Transcript levels upon knockdown of *clpP2X* with *pcnB2* complementation. Data displayed are the values from three independent biological replicates of triplicate RT-qPCRs. Values are normalized to the 10-h uninduced ClpP2 value for each experiment. Samples were induced using 10 nM aTc. (C) IFU titers following induction of knockdown and complementation at 4 hpi. Values are presented on a log_10_ scale percentage of the respective uninduced titer. (D) Plasmid retention of *clpP2X* knockdown with *pcnB2* complementation. Download FIG S4, PDF file, 2.5 MB. Copyright © 2020 Wood et al. 2020 Wood et al. This content is distributed under the terms of the Creative Commons Attribution 4.0 International license.

When dCas9 expression was induced at 10 hpi in the various transformants, we observed a marked and rapid decrease in *clpP2*, *clpX*, and *pcnB2* transcript levels compared to that in the uninduced controls at 14 hpi (Fig. 5C). Importantly, we did not observe a decrease in transcript levels for unrelated genes associated with different stages of the developmental cycle (*clpP1*, *euo*, and *omcB*) (Fig. S2) (12, 17, 41, 42). We also observed abnormal inclusion morphology with individual organisms aggregated to one side of the inclusion (Fig. 5A). Complementation with ClpP2_FLAG resulted in decreased *clpX* transcripts compared to that in the uncomplemented strain but an increased level of *clpP2* transcripts, as expected (Fig. 5F). However, ClpP2 complementation did not fully restore inclusion morphology, as organisms still displayed some aggregation within the inclusion, albeit intermediate between the uncomplemented knockdown and control conditions (Fig. 5B). Complementation with PcnB2_FLAG did not impact *clpP2* and *clpX* transcript levels during knockdown but did increase *pcnB2* transcripts approximately 10-fold relative to that in the uninduced samples (Fig. S4B). Abnormal inclusion morphology was also evident (Fig. S4A). As previously observed, IncA expression was uniformly blocked after dCas9 induction (Fig. S3) (38). We also attempted a similar *clpX* complementation strategy but saw no effect on *clpP2* or *pcnB2* transcript levels during knockdown (unpublished observation). Given that we tagged our dCas9 variant with an SsrA recognition motif and that ClpX recognizes the SsrA tag (see below), the dCas9 was likely targeted for degradation by the plasmid-encoded ClpX, subsequently eliminating any knockdown.

We next assayed chlamydial growth as measured by IFU recovery and chlamydial morphology by immunofluorescence after inducible knockdown of the target genes. Expression of dCas9 was induced at 4 hpi, and IFUs were harvested at 24 and 48 hpi and the titers were determined on fresh cell monolayers in the presence of penicillin, the selection agent. When *clpP2X* expression was blocked at 4 hpi, we noted a 5-fold decrease in penicillin-resistant (i.e., transformants containing the CRISPRi plasmid) IFUs at 24 hpi but a >200-fold decrease at 48 hpi (Fig. 5D). Immunofluorescence assay images supported this drop in viable organisms, revealing a severely diminished number of organisms within the inclusion (Fig. 5A). When performing these assays in the presence of penicillin, we also observed numerous penicillin-sensitive organisms (i.e., aberrant RBs [43]) during the titration step, suggesting that the plasmid conferring resistance and encoding the CRISPRi system was being lost after induction of dCas9 expression. To test this, we quantified plasmid retention in the *clpP2X* knockdown samples and observed that blocking *clpP2X* expression resulted in ∼75% plasmid loss at 24 hpi and greater than 90% loss at 48 hpi (Fig. 5E), which was closely recapitulated with *pcnB2* complementation (Fig. S4C and D). These effects on IFUs and plasmid retention were not observed for *incA* knockdown (Fig. S3B and C). We note that *incA* knockdown did result in a reproducible, but transient, increase in IFUs at 24 hpi that returned to “normal” levels at 48 hpi (Fig. S3B). The reasons for this are not clear. Nonetheless, we conclude from these data that blocking *clpP2X* expression is deleterious to *Chlamydia*, further highlighting its essentiality to this pathogen. Interestingly, complementation with *clpP2* largely rescued the drop in recoverable IFUs while maintaining plasmid loss compared to that under the uncomplemented condition (Fig. 5G and H). This suggests that much of the loss in bacterial viability during *clpP2X* knockdown is attributable to reduced ClpP2 levels.

### ClpX function is required for degradation of an SsrA-tagged substrate in C. trachomatis.

Recently, ClpX-specific inhibitors were synthesized by the Sieber group and were shown to interfere with ClpX ATPase activity (44). One compound, identified as 334, was shown to have potent anti-ClpX activity, whereas a derivative, 365, was inactive. We performed *ab initio* modeling and molecular dynamics simulations (45) to determine if these compounds could interact with an ADP-bound hexameric ClpX_Ctr_. For 334, a high scoring model (−9.1 kcal/mol binding affinity, root mean square deviation [RMSD] ∼ 0) was predicted with the drug binding near the ATP binding pocket, suggesting a mechanism of action where 334 likely occludes the ATPase site (see Fig. S5). Whether the effect stems from the blocking of ATP binding and subsequent destabilization of the complex, attenuation of ATPase function by preventing a conformational change of the complex, or steric hindrance of complex formation remains to be elucidated. Conversely, compound 365 bound outside the ATP pocket with a much lower score (see Fig. S6).

10.1128/mBio.02016-20.5 FIG S5 Docking simulation of the ClpX inhibitor 334 on a ClpX model. (A) PDB structure of 334. Inset is the two-dimensional (2D) structure of the drug. (B) Ribbon model of docked 334 within the ClpX hexamer. The best scoring model is shown. Only the two ClpX subunits contacting the model are shown (A in gray, B in seafoam green). The Walker A motif (red), Walker B motif (purple), sensor 1 motif (dark green), and sensor 2 motif (orange) of subunit A are colored for visualization. The arginine finger is labeled light green of subunit B. The center picture is oriented as outside the complex looking inward, and the other images are rotations as indicated. (C) Surface rendering of the ClpX subunits with docked 334 are shown with coloration as for panel B. Download FIG S5, PDF file, 2.7 MB. Copyright © 2020 Wood et al. 2020 Wood et al. This content is distributed under the terms of the Creative Commons Attribution 4.0 International license.

10.1128/mBio.02016-20.6 FIG S6 Docking simulation of the ClpX inhibitor 365 on a ClpX model. (A) PDB structure of 365. Inset is the 2D structure of the drug. (B) Ribbon model of docked 365 within the ClpX hexamer. The best scoring model is shown. Only the two ClpX subunits contacting the model are shown (A in gray, B in seafoam green). The Walker A motif (red), Walker B motif (purple), sensor 1 motif (dark green), and sensor 2 motif (orange) of subunit A are colored for visualization. The arginine finger is labeled light green of subunit B. The center picture is oriented as outside the complex looking inward, and the other images are rotations as indicated. (C) Surface rendering of the ClpX subunits with docked 365 are shown with coloration as for panel B. Download FIG S6, PDF file, 2.9 MB. Copyright © 2020 Wood et al. 2020 Wood et al. This content is distributed under the terms of the Creative Commons Attribution 4.0 International license.

**FIG 6:**
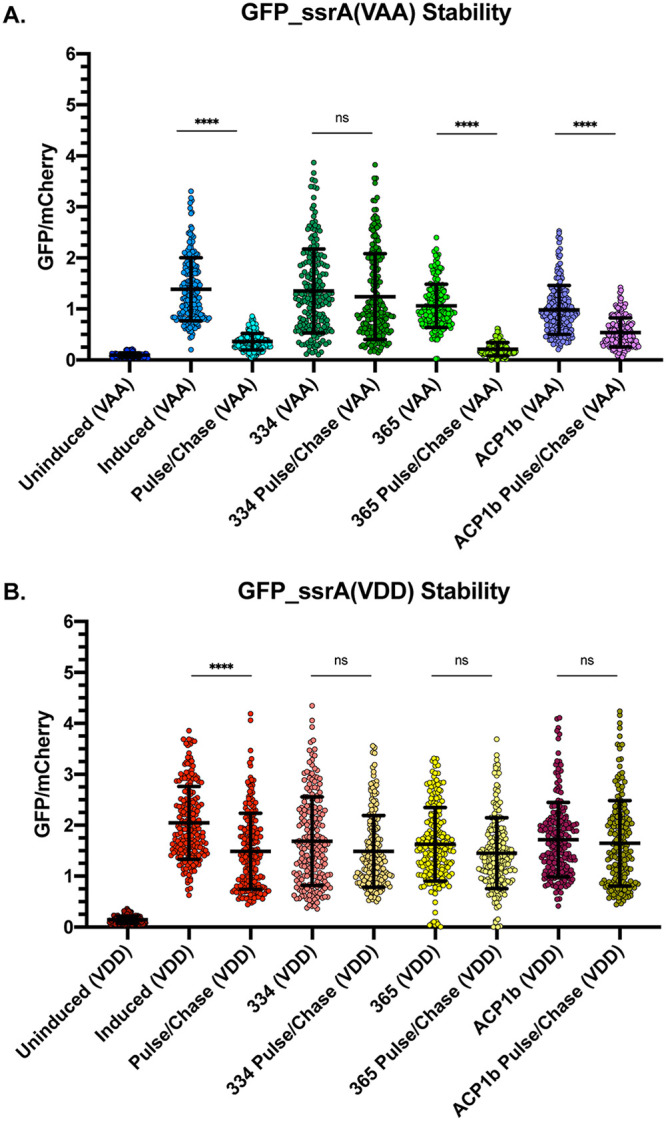
Chemical disruption of ClpX function protects SsrA-tagged GFP. (A) The ratio of integrated density measurements of GFP to mCherry for SsrA(VAA)-tagged GFP. All samples were induced or not at 8 hpi with 20 nM aTc. Media were replaced with or without drug at 16 hpi, and aTc induction was maintained or removed (for pulse-chase samples). Samples were fixed at 20 hpi, and GFP and mCherry intensity were quantified using Fiji. ****, *P* ≤ 0.0001; ns, not significant by ordinary one-way ANOVA. (B) Same conditions as for panel A but with GFP(VDD).

Given the predicted binding of the ClpX inhibitors on the structure of ClpX_Ctr_, we sought to determine whether these drugs effectively blocked ClpX function *in vivo*. We utilized a chlamydial SsrA-tagged green fluorescent protein (GFP) variant [GFP(VAA)], which was previously shown to be a substrate of ClpX_Ctr_ *in vitro* (46), or GFP tagged with a mutated SsrA tag [GFP(VDD)]. When mutating VAA to VDD, GFP is rendered resistant to ClpX recognition (47). Using an anhydrotetracycline-inducible pBOMB4 derivative encoding constitutive mCherry as a metric of chlamydial growth (here referred to as pBOMBmC), we cloned each GFP variant into pBOMBmC and transformed these constructs into C. trachomatis L2. Samples were infected, and GFP expression was induced at 8 hpi. All induced samples were then allowed to develop until 16 hpi, when the medium was replaced by either Dulbecco’s modified Eagle’s medium (DMEM) only or DMEM containing the designated antibiotic (Fig. 6A and B; see also Fig. S7). Immediately following drug addition, induction of GFP expression was maintained or not, where removal of anhydrotetracycline (aTc) was denoted by pulse-chase. GFP signal was normalized to the constitutive mCherry signal expressed on the plasmid backbone. We observed that removal of inducer results in rapid loss of GFP(VAA) but not GFP(VDD) signal compared to continuous induction, indicating that GFP(VAA) is degraded as expected (Fig. 6A and B; Fig. S7). Treatment with compound 334 resulted in sustained GFP(VAA) signal, consistent with its documented effects on ClpX (44). Importantly, compound 365, the inactive 334 derivative, and ACP1b, a ClpXP uncoupling drug with a mechanism of action similar to that of the acyldepsipeptide (ADEP) compounds (17, 48), failed to protect GFP(VAA) from degradation (Fig. 6A), which further supports the specificity of compound 334 in mediating this effect. We then tested the effect of these drugs on chlamydial viability and noted a multiple log_10_ decrease in recoverable IFUs (see Fig. S8), supporting that blocking ClpX function is highly detrimental to *Chlamydia*. However, in attempting to assess the effect of these compounds on *Chlamydia*, we noted reduced host cell viability with prolonged treatment (Fig. S8D), making interpretation of those data unclear.

10.1128/mBio.02016-20.7 FIG S7 Chemical disruption of ClpX function blocks degradation of an SsrA-tagged substrate in C. trachomatis L2. Representative immunofluorescence images corresponding to data presented in Fig. 6 of the main text. Representative images are of one field of view of one biological replicate. All samples were induced or not at 12 hpi with 20 nM aTc. Media were replaced with or without drug at 16 hpi, and aTc induction was maintained or removed (for pulse-chase samples). Samples were fixed at 20 hpi and were imaged as previously described using the same exposure time for all samples. Bars, 20 μm. Individual color channels were gray scaled and inverted to give clear distinction of fluorescent protein intensity. Download FIG S7, PDF file, 0.1 MB. Copyright © 2020 Wood et al. 2020 Wood et al. This content is distributed under the terms of the Creative Commons Attribution 4.0 International license.

10.1128/mBio.02016-20.8 FIG S8 Chemical disruption of ClpX function is highly detrimental to *Chlamydia*. (A) Immunofluorescence assay (IFA) at 24 h postinfection (hpi). All drugs added at a final concentration of 25 μg/ml. Drugs were added at 8 hpi for the 24-h treatment samples. Drugs were added 15 min postinfection and removed at 8 hpi for the 8-h time of infection pulse samples. MOMP is stained in green, and DNA is stained with DAPI. Images were acquired on a Zeiss LSM800 microscope at ×63 magnification. Bar, 10 μm. (B) IFA of 48-hpi samples. Samples stained for MOMP (green) and DNA (blue). Bar, 10 μm. Drug was added at 8 hpi for 48-h and reactivation samples. Medium removed, drug washed out, and medium with drug added back at 24 h for 48-h samples. For reactivation samples, medium replaced with DMEM, no drug. (C) Recoverable inclusion forming units (IFUs) from the indicated conditions. Totals presented as log_10_ IFUs recovered. Standard deviations are displayed on graphs as error bars. (D) PrestoBlue cell viability assay upon drug treatment from 8 to 24 hpi. Values are displayed as percentages of the untreated samples. UTD, untreated. Download FIG S8, PDF file, 0.1 MB. Copyright © 2020 Wood et al. 2020 Wood et al. This content is distributed under the terms of the Creative Commons Attribution 4.0 International license.

## DISCUSSION

Given the unique roles and protein repertoires of the chlamydial developmental forms (EB/RB), we hypothesize that protein degradation is a critical factor in the process of differentiation from one form to the other. The Clp system is highly conserved in both prokaryotic and eukaryotic systems, where it has been described to perform important functions in both proteostasis and pathogenesis (49). The Clp system is nominally composed of a proteolytic subunit, ClpP, and an AAA+ ATPase that functions as an unfoldase to recognize substrates and feed them into the ClpP barrel for degradation (23). The work presented here expands our understanding of the Clp protease system of an obligate intracellular bacterial pathogen, Chlamydia trachomatis. Focusing on characterization of ClpX_Ctr_ and the function of the *clpP2X* operon, we demonstrated the importance of the ClpXP protease system during chlamydial growth and development.

Multiple lines of evidence support that the chlamydial ClpX is a bona fide AAA+ ATPase. First, multiple-sequence alignment of ClpX_Ctr_ to orthologs of other bacteria revealed a perfect conservation of the motifs involved in nucleotide binding, ATP hydrolysis, and nucleotide-state sensing (Fig. 1) (50, 51). Second, homology-directed and *ab initio* modeling of ClpX_Ctr_ revealed that the spatial orientation of these domains is conserved as well (Fig. 1), though we acknowledge that structural studies are critical to drawing conclusions about ClpX_Ctr_ conformational states. Third, ClpX_Ctr_ interacts with itself to form a homohexamer that possesses ATPase activity (Fig. 2). Importantly, this ATPase activity was disrupted by a targeted mutation in the Walker B motif while having no effect on the oligomerization properties of the protein. Fourthly, a characterized ClpX inhibitor that disrupts its ATPase activity also blocked SsrA-mediated degradation *in vivo* and disrupted the growth of C. trachomatis serovar L2 (Fig. 6; see also Fig. S7 in the supplemental material). Finally, overexpression of a ClpX_Ctr_ ATPase mutant *in vivo* negatively impacted chlamydial growth and development (Fig. 3 and 4).

While we have characterized the ATPase function of ClpX_Ctr_ and its role in chlamydial growth, further work remains to determine whether this ClpX ortholog functions as an unfoldase. Nevertheless, our bioinformatics analysis supports this, as ClpX_Ctr_ retains substrate recognition motifs, including both pore loops and the RKH motif for the gripping and translocation of substrates (27–31). *Chlamydia* spp. also harbor the transfer-messenger RNA (tmRNA)/SsrA tagging system for ribosomal rescue (18, 52–55), which fits a model where ClpX_Ctr_ may play an integral role in turnover of tagged partially translated peptides. A recent article, using an *in vitro* assay for SsrA-tagged GFP degradation, suggests this function of chlamydial ClpX may be conserved (46). Our *in vivo* data exhibiting SsrA-tagged GFP protection with the addition of a ClpX inhibitor also suggests ClpX_Ctr_ can target SsrA-tagged substrates, but whether this tagging is for ribosomal rescue or more specific purposes (56, 57) remains to be determined and is currently under investigation by our research group.

In *Chlamydia*, *clpX* is located in an operon with *clpP2*. Our data indicate that, not surprisingly, the ClpP2X_Ctr_ system is highly regulated and essential. We previously demonstrated that treatment with ClpP-targeting antibiotics severely impacts the growth of *Chlamydia* (17). Here, we performed a systematic analysis of the effects of overexpression of wild-type or inactivated ClpP2X_Ctr_ components. The overexpression of wild-type ClpP2_Ctr_ and/or ClpX_Ctr_ had no biologically or statistically significant effect on chlamydial growth that we could measure. However, extended overexpression of mutant ClpP2_Ctr(S98A)_ and/or ClpX_Ctr(E187A)_ resulted in abrogation of chlamydial growth as measured by the recovery of infectious progeny. Three observations should be noted. First, the effect of inducibly expressed proteins was measured in the presence of the endogenous chromosomally expressed copies of these proteins. Therefore, it is likely that the mutants would have even more dramatic effects on chlamydial growth and that disruption of these components results in lethality. For ClpX_Ctr_, this is supported by the effects of the ClpX inhibitor on *Chlamydia* (Fig. S8), which effectively stopped chlamydial growth. Second, we demonstrated that the mutant proteins were able to interact *in vitro* with wild-type isoforms (Fig. 2) (17). Therefore, we can infer that overexpression of the mutant proteins leads to their incorporation into the endogenous Clp machinery to disrupt or impair its function. Third, to our knowledge, ours is the first study to ectopically express two different tagged proteins in *Chlamydia*, showing both the feasibility of this approach and its potential utility to dissect chlamydial biology.

The overexpression of the catalytically inactive mutant Clp proteins in *Chlamydia* revealed potentially subtle differences in the role of each component in chlamydial growth and development. Surprisingly, we noted a roughly 50% reduction in detectable genomes (Fig. 4A) when ClpX_Ctr(E187A)_ was expressed, whereas IFUs were reduced roughly 20-fold (Fig. 3). The production of EBs as measured by HctB levels did not appreciably change (Fig. 4B and C). This suggests that, while development is hindered, the drop in IFUs may be due to defective EB viability, infectivity, or inclusion establishment and not a reduction in secondary differentiation *per se*. Support for this comes from electron microscopy images, which revealed unusual morphologies after overexpression of the mutant ClpX_Ctr_ isoform (Fig. 4D; Fig. S1). As noted above, the SsrA-mediated degradation of partially translated products may be critical during the differentiation process. As RBs condense to EBs, DNA replication, transcription, and translation are significantly abrogated, and we hypothesize that those partially translated proteins trapped during this differentiation step require degradation. This may explain the effects we observed on decreased EB viability during overexpression of the ClpX mutant isoform. Whether the degradation of such partial peptides occurs during RB-to-EB differentiation or during the next infection when an EB differentiates to an RB remains to be investigated.

**FIG 7:**
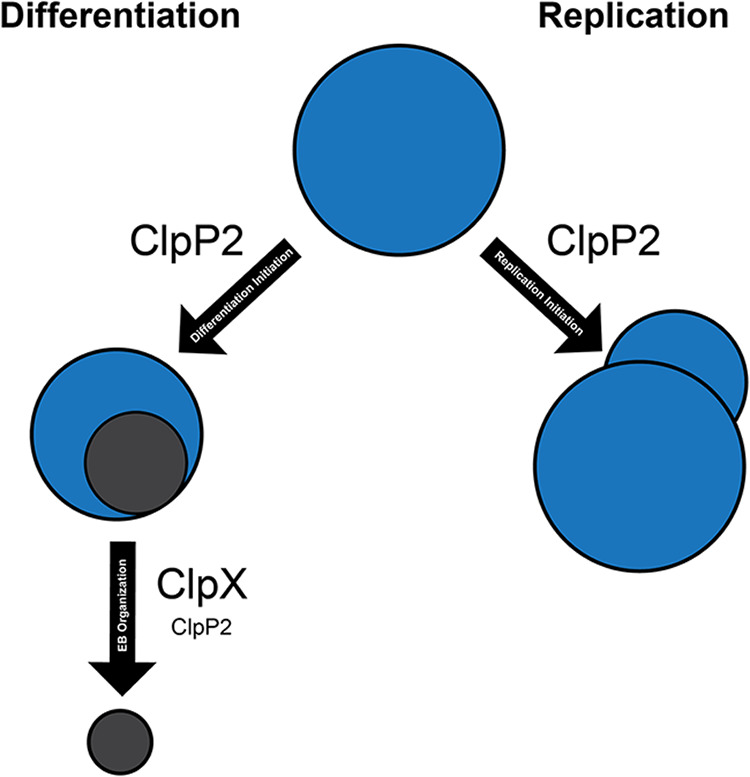
Proposed model for ClpP2X function in *Chlamydia*. An RB ultimately has two fates: differentiation or replication. Based on our data showing the impact of ClpP2 disruption on the developmental cycle, we hypothesize that ClpP2 may function in triggering either event based on its degradative target at either time point. Furthermore, we posit that ClpX serves an important function in EB organization, given that ClpX affects recoverable IFUs without reducing the amount of HctB produced.

Conversely, for ClpP2_Ctr(S98A)_ overexpression, the substantial IFU decrease coupled with a sharp drop in gDNA levels indicate that ClpP2_Ctr_ plays a role in developmental cycle progression. HctB levels were also significantly reduced, which is consistent with the lack of EB generation. Taken together, these data may indicate that ClpP2_Ctr_ is integral to developmental cycle progression or differentiation and that its function is tightly regulated. We cannot, however, conclude that secondary differentiation is directly affected due to the fact that total organism numbers were severely reduced. Rather, our proposed model suggests that ClpP2_Ctr_ disruption may affect both factors by a mechanism that we are currently working to identify. Conversely, ClpX_Ctr_ may serve a more prominent ClpP2_Ctr_-independent function in differentiation of the organism (Fig. 7). Interestingly, several studies on the periplasmic protease HtrA in *Chlamydia* have implicated this protease in membrane reorganization (58–60). Therefore, one model may be that the ClpXP system is required for cytosolic turnover whereas HtrA is required for membrane reorganization during differentiation.

We successfully generated chlamydial transformants with an inducible knockdown system to repress ClpP2X_Ctr_ expression. To date, this study is the first of its kind in *Chlamydia* to knock down genes that are essential, highlighting the utility of CRISPRi in studies of chlamydial biology while providing insight into possible ClpP2X_Ctr_ function. We also demonstrated that these knockdowns may be complemented by insertion of the gene of interest under the inducible promoter as a transcriptional fusion with the dCas9 (Fig. 5 and data not shown). Notably, we observed a large decrease in IFU production coupled with an increase in plasmid loss after inhibition of *clpP2X* expression (Fig. 5). These effects were not observed when targeting a nonessential gene. Additionally, we demonstrated that the impact on IFUs, but not the effect on plasmid retention, was reversed by complementation. Of note, penicillin does not kill chlamydiae but blocks cell division (61, 62), which keeps the organism transcriptionally in an RB-like state (63). This suggests that knocking down an essential gene(s) puts selective pressure on the chlamydiae to lose the plasmid encoding the CRISPRi system. This has important ramifications for long-term experiments and functional analyses. Nevertheless, the CRISPRi system represents a significant advance for our ability to study essential systems in this obligate intracellular bacterium.

A recent *in vitro* study suggested that homoheptamers of ClpP1 form a complex with homoheptamers of ClpP2 that interact with ClpX (46), yet we observed distinct differences when overexpressing mutant isoforms of these proteins in *Chlamydia* (17). If these ClpP proteins also interact with the other chlamydial AAA+ ATPase, ClpC, then it is not surprising that we observed differences between ClpP2 and ClpX in our assays, since disrupting ClpP2 could also be impacting ClpC function. This would also beg the question why we did not observe similar impacts on chlamydial growth when overexpressing each mutant isoform (17). One possibility may be that ClpX interacts with ClpP2 in a ClpP1P2 complex, whereas ClpC interacts with ClpP1 in a ClpP1P2 complex. In this scenario, disrupting ClpP2 would act epistatically to ClpX. This is not inconsistent with our data and proposed model. Clearly, the composition of the Clp complexes remains to be determined *in vivo*, and this is an area of ongoing investigation.

In conclusion, we have demonstrated the importance of the ClpP2X_Ctr_ system to chlamydial development, but many questions remain unanswered. These include why ClpP2_Ctr_ and ClpX_Ctr_ may serve independent purposes and what substrates this system may be targeting. Additionally, we need to identify any cofactors, chaperones, adaptor proteins, or a lack thereof that may be pertinent to this system. We plan to dissect the structural motifs of ClpP2_Ctr_ and ClpX_Ctr_ to determine if any of the noted differences from other bacterial Clp proteins alter activity, which may aid in our goal of further functional assessment. Finally, continued experimentation to address our overarching hypothesis that protein turnover is critical to chlamydial differentiation, and that the Clp system is a key mediator of this process, is required. Overall, we conclude that the chlamydial ClpP2X_Ctr_ system is critical to the development of these obligate intracellular bacteria.

## MATERIALS AND METHODS

### Strains and cell culture.

The human epithelial cell line HEp-2 was used for the overexpression assays, gDNA and protein extractions, and antibiotic studies. McCoy mouse fibroblasts were used for chlamydial transformation, and human epithelial HeLa cells were used for plaque purification. All of these cell lines were passaged routinely in Dulbecco’s modified Eagle’s medium (DMEM; Gibco/Thermo Fisher) and 10% fetal bovine serum (FBS; Sigma, St. Louis, MO) and verified to be mycoplasma-free using a LookOut Mycoplasma PCR Detection kit (Sigma). Density gradient-purified Chlamydia trachomatis L2/434/Bu (ATCC VR902B) EBs were used for the antibiotic studies. C. trachomatis serovar L2 EBs (25667R) naturally lacking the endogenous plasmid were prepared and used for transformation (see reference 64).

### Bioinformatics analysis.

Gene sequences of Chlamydia trachomatis were obtained from STDGen database (http://stdgen.northwestern.edu) or KEGG Genome Browser (65–67). RefSeq protein sequences from Escherichia coli, Bacillus subtilis, Mycobacterium tuberculosis, Staphylococcus aureus, and Pseudomonas aeruginosa were acquired from the NCBI protein database (https://www.ncbi.nlm.nih.gov/guide/proteins/). ClpX pairwise protein alignments to find sequence identity were performed using the NCBI Protein BLAST function (https://blast.ncbi.nlm.nih.gov/Blast.cgi) (68). Multiple-sequence alignments were performed using Clustal Omega (69) with default settings and were presented using Jalview Version 2 (70). PDB files for predicted monomeric three-dimensional (3D) structures were acquired from the Phyre2 website (http://www.sbg.bio.ic.ac.uk/phyre2/html/page.cgi?id=index) (71). Complexes were modeled using SWISS-MODEL available on the ExPASy server (72–75). Protein models and model alignments were rendered using the UCSF Chimera package from the Computer Graphics Laboratory, University of California, San Francisco (supported by NIH P41 RR-01081) (76). Docking analyses were performed using AutoDock Vina (45). Molecules were prepped using Dunbrack rotamer libraries (77, 78) to replace incomplete side chains and ANTECHAMBER for charge assignment and topology generation (79).

### Plasmid construction.

A full list of the primers and plasmids used is included in Table S1 in the supplemental material. The Gateway recombination system of cloning was used for plasmids for the bacterial adenylate cyclase two-hybrid (BACTH) system (80). The genes were amplified from Chlamydia trachomatis L2 genomic DNA with added *attB* recombination sites. The PCR products were then incubated with a pDONR 221 entry vector (containing *attP* recombination sites) in the presence of BP Clonase II (Invitrogen) to insert the gene via flanking *attP* recombination sites and remove the *ccdB* insert, resulting in an entry vector containing the gene of interest flanked by *attL* sites. These constructs were transformed into DH5α chemically competent E. coli and plated onto kanamycin-containing LB agar. Plasmid was isolated and used for the LR reaction into one of three destination vectors (pST25-DEST, pSNT25-DEST, or pUT18C-DEST). The same entry vector for any given gene was used for all three LR reactions to insert into the destination vector. Entry vector and destination were incubated at a 1:1 ratio. DH5α E. coli cells were transformed with 2 μl of the reaction mix. Purified plasmid from an individual colony was sequence verified prior to use in the BACTH assay (see below).

10.1128/mBio.02016-20.10 TABLE S1 List of primers, plasmids, and strains used in this study. Download Table S1, DOCX file, 0.1 MB. Copyright © 2020 Wood et al. 2020 Wood et al. This content is distributed under the terms of the Creative Commons Attribution 4.0 International license.

Constructs for chlamydial transformation were created using the HiFi Cloning (New England BioLabs) protocol. Primers were designed to add a poly-histidine (6×His) tag to the gene of interest with the overlap to insert into the shuttle vector. Primers were generated using the NEBuilder assembly tool available from New England BioLabs (http://nebuilder.neb.com). The backbone used was the pTLR2 derivative of the pASK plasmid (81). For the CRISPRi plasmid, the S. aureus dCas9 was PCR amplified from pX603-AAV-CMV::NLS-dSaCas9(D10A,N580A)-NLS-3xHA-bGHpA (a gift from F. Zhang; Addgene plasmid number 61594 [39]) and inserted into a derivative of pBOMB4-Tet::L2 (kind gift of T. Hackstadt, NIH [82]) modified to weaken its ribosome binding site (data not shown). The gRNA cassettes were designed as previously described (38), ordered as gBlock fragments from IDT (Coralville, IA), and inserted into the BamHI site of the pBOMB4-Tet derivative encoding *Sa_dCas9* to produce, for example, the plasmid pBOMBLCRia(*clpP2X*)::L2. HiFi reactions were assembled according to the manufacturer’s protocol. The reaction was transformed into DH10β E. coli, and isolated plasmid was verified by restriction enzyme digest and sequencing by Eurofins Genomics. Sequence-verified plasmids were transformed into Δ*dam* Δ*dcm* E. coli (New England BioLabs) to produce demethylated plasmid, which was verified as described earlier prior to transformation into C. trachomatis (see below).

For mutation of ClpX Walker B motif, Q5 mutagenesis (New England BioLabs) was used. Primers were designed encoding the E187A mutation for PCR linearization of the plasmid. ClpX BACTH constructs were used as a template for the PCR amplification, and plasmids were recircularized by kinase-ligase-DpnI (KLD) reaction. The resulting reactions were transformed into DH5α E. coli for plasmid production. Plasmids were isolated, and mutations were verified by Sanger sequencing (Eurofins Genomics) prior to use in the BACTH system. These plasmids also served as the template for the PCRs to produce PCR products for insertion of the mutant *clpX* gene into the pTLR2 plasmid.

Strains created or used in this study are listed in Table S1. Transformed E. coli strains were maintained on LB agar plates, with antibiotics as necessary. To extract chlamydial genomic DNA, EBs were subjected to heat and proteinase K treatment prior to phenol-chloroform extraction (83). Sodium hydroxide lysis was utilized for the extraction of E. coli genomic DNA. For cloning into the pLATE31 plasmid, the aLICator LIC cloning and expression kit 3 (Thermo Scientific) was used according to the manufacturer’s specifications. Plasmids were first cloned into DH5α E. coli for plasmid propagation. Transformants were screened for inserts using colony PCR with Fermentas master mix (Thermo Scientific), and positive clones were grown for plasmid isolation (GeneJet Plasmid Miniprep kit; Thermo Scientific). Sequence-verified plasmids were then transformed into BL21(DE3) Δ*clpPAX* E. coli (46) for subsequent protein purification.

### Purification of recombinant ClpX.

His-tagged C. trachomatis ClpX and C. trachomatis ClpX_E187A_ were purified from 500-ml cultures of BL21(DE3) Δ*clpPAX* E. coli transformed with the respective plasmid based on the protocol described in reference 17. Samples were induced with 0.5 mM isopropyl-β-d-thiogalactopyranoside (IPTG) and incubated with shaking for 20 h at 18°C. Cultures were pelleted and frozen at −80°C prior to purifications. Samples were suspended in buffer A (25 mM Tris base [pH 7.5], 300 mM NaCl, and 10 mM imidazole), sonicated, bound to HisPur cobalt resin (Thermo Scientific), and washed in buffer A. Proteins were eluted from the resin using buffer B (25 mM Tris base [pH 7.5], 300 mM NaCl, and 300 mM imidazole). Buffer exchange for ATPase assay buffer (25 mM HEPES [pH 7.2], 200 mM KCl, 20 mM MgCl_2_, and 10% glycerol) was performed using a Millipore Amicon Ultra 15 filtration unit (3-kDa cutoff). ClpX proteins were quantified using the Bio-Rad protein assay, assessed for purity on 10% SDS-PAGE gels with Coomassie staining (see Fig. S9), and identified using anti-His-tag Western blotting. Blotting was performed using a mouse monoclonal anti-6×His antibody (1:1,000, Millipore HIS.H8) and a goat anti-mouse IgG horseradish peroxidase (HRP)-conjugated secondary antibody (1:2,000). Protein samples were aliquoted and stored at −80°C.

10.1128/mBio.02016-20.9 FIG S9 Example ClpX protein purifications. Recombinant 6× His-tagged ClpX and ClpX_E187A_ were purified using cobalt-based immobilized metal affinity chromatography. Samples were quantified, and 1- and 5-μg aliquots were run on 10% SDS-PAGE followed by staining with Coomassie brilliant blue. Three ClpX and two ClpX_E187A_ purifications were performed using BL21(DE3) Δ*clpPAX* E. coli. Download FIG S9, PDF file, 0.8 MB. Copyright © 2020 Wood et al. 2020 Wood et al. This content is distributed under the terms of the Creative Commons Attribution 4.0 International license.

### *In vitro* analysis of ClpX homo-oligomerization.

Ten micrograms of purified protein was incubated at for 20 min at 37°C in oligomerization buffer (25 mM Tris base [pH 7.5], 5 mM KCl, 5 mM MgCl_2_, 1 mM dithiothreitol [DTT], and 1% glycerol) prior to mixing with a 5× native sample buffer (5 mM Tris [pH 6.8], 38 mM glycine, 0.06% bromophenol blue). Assays were analyzed on a Bio-Rad MiniProtean 4% to 20% gradient gel for native PAGE. Gels were assessed using Coomassie staining.

### Assessment of ClpX ATPase activity *in vitro*.

A 49.5-μl reaction mixture containing 1.5 μg of recombinant wild-type ClpX or ClpX_E187A_ in ATPase assay buffer (see above) supplemented with 1.7% dimethyl sulfoxide (DMSO) was preincubated for 10 min at room temperature without ATP. Next, ATP dissolved in ATPase assay buffer was added to 1 μM, giving a final volume of 50 μl, and the reaction mixture was incubated at 30°C for 1.5 h. After the 1.5 h, the reaction mixtures were incubated for an additional 30 min at room temperature. Fifty microliters of Kinase-Glo reagent (Promega) was then added and incubated at room temperature for 10 min. Luminescence of the reaction, reflecting ATP not consumed by ClpX or ClpX_E187A_, was then measured using a BioTek Synergy H1 plate reader. Reactions were performed in duplicates at least three times with at least two independent protein preparations.

### Determining protein-protein interactions with the BACTH system.

The bacterial adenylate cyclase two-hybrid (BACTH) assay was utilized to test the interaction between ClpX wild-type and the mutant (84). The genes of interest are translationally fused to one of either subunit, denoted as T18 and T25, of the Bordetella pertussis adenylate cyclase toxin, which can complement adenylate cyclase deficient (Δ*cya*) DHT1 E. coli. Wild-type and mutant *clpX* genes cloned into one of the pST25, pSNT25, or pUT18C Gateway vectors was tested for both homotypic and heterotypic interactions (9, 80). Plasmids from each background were cotransformed into chemically competent DHT1 E. coli cells, which were plated on a double antibiotic minimal M63 medium selection plate supplemented with 0.5 mM IPTG for induction of the protein, 40 μg/ml 5-bromo-4-chloro-3-indolyl-β-d-galactopyranoside (X-Gal), 0.04% casein hydrolysate, and 0.2% maltose. Leucine zipper motifs were used for controls in pKT25 and pUT18C backgrounds on the appropriate antibiotic selection plates because these have been previously shown to interact (85). Blue colonies, indicative of positive interaction, were screened using the β-galactosidase assay. Random positive colonies were selected and grown in M63 minimal medium with the appropriate antibiotics; 0.1% SDS and chloroform were used to permeabilize the bacteria prior to addition of 0.1% *o*-nitrophenol-β-galactoside (ONPG), and 1 M NaHCO_3_ was used to stop the reaction after precisely 20 min of incubation at room temperature. Absorbance at the 405 nm wavelength was recorded and normalized to bacterial growth (optical density of 600 nm [OD_600_]), dilution factor, and time (in minutes) of incubation prior to stopping the reaction. Totals were reported in relative units (RU) of β-galactosidase activity.

### Chlamydial transformation.

The protocol followed was a modification of the method developed by Mueller et al. (86) and as previously described (17). For transformation, 10^6^ C. trachomatis serovar L2 EBs (25667R) naturally lacking the endogenous plasmid were incubated with 2 μg of unmethylated plasmid in a volume of 50 μl CaCl_2_ at room temperature for 30 min. Reaction volume was sufficient for one well of a six-well plate of McCoy mouse fibroblasts. Transformants were mixed with 1 ml of Hanks balanced salt solution (HBSS) and added to 1 ml of HBSS in a six-well plate. The plates were centrifuged at room temperature for 15 min at 400 × *g*. The plate was then incubated at 37°C for 15 min. After incubation, the HBSS was aspirated and replaced with antibiotic-free DMEM plus 10% FBS. Eight hours postinfection, the medium was replaced with DMEM containing 1 μg/ml cycloheximide and 1 U/ml penicillin. Cells infected with transformants were passaged every 48 h until a population of penicillin-resistant bacteria was established. EBs were harvested and frozen in sucrose-phosphate (2SP) (64) solution at −80°C.

### Determining the effect of overexpression of wild-type and mutant Clp proteins via immunofluorescence and inclusion-forming unit analysis.

C. trachomatis transformants containing plasmids encoding the 6×His-tagged protein of interest were used to infect a confluent monolayer of HEp-2 cells. Penicillin treatment was maintained throughout the duration of the infection. At 10 hpi, samples were induced or not with 10 nM anhydrotetracycline (aTc). At the designated time points, three wells of a 24-well plate were scraped in 2SP, vortexed with three 1-mm glass beads, and frozen at −80°C. At the same time point, a coverslip was fixed in 3.25% formaldehyde and 0.025% glutaraldehyde for 2 min, followed by permeabilization with cold 90% methanol for 1 min. Coverslips were labeled with primary goat anti-major outer membrane protein (MOMP; Meridian, Cincinnati, OH), rabbit anti-6×His (Abcam, Cambridge, MA), and 4′,6-diamidino-2-phenylindole (DAPI). Appropriate donkey secondary antibodies were used (Invitrogen, Carlsbad, CA). Images were acquired on an Axio ImagerZ.2 equipped with Apotome.2 optical sectioning hardware and X-Cite Series 120PC illumination lamp. Frozen IFU samples were titrated onto a fresh monolayer of HEp-2s without antibiotics. At 24 hpi, samples were fixed with methanol for 10 min, stained for MOMP, and enumerated.

### Genomic DNA isolation and qPCR enumeration of genomic equivalents.

At 24 or 48 hpi, one well of a six-well plate was scraped into the medium overlay and pelleted at 17,000 × *g* at 4°C for 15 min. Each sample was resuspended in 500 μl of cold phosphate-buffered salin (PBS), frozen three times at −80°C, and processed using the Qiagen DNeasy blood and tissue kit according to the manufacturer’s specifications. DNA concentrations were assessed using a spectrophotometer prior to dilution down to 5 ng/μl. Five microliters of the resulting dilution was used for a 25-μl quantitative PCR (qPCR) volume using SYBR green PCR master mix (Applied Biosystems). Each reaction was performed in triplicates. A standard curve using C. trachomatis L2 genomic DNA was generated for interpolation of sample threshold cycle (*C_T_*) values. This experiment was performed three times for three biological replicates.

### Analysis of HctB levels upon Clp overexpression.

At 24 or 48 hpi, one well of a six-well plate per test condition was rinsed twice with HBSS. To lyse the cells, 500 μl of denaturing lysis buffer (8 M urea, 10 mM Tris, 2.5% 2-mercaptoethanol, 1% SDS) was added to each well and incubated for 15 min at room temperature. Three hundred units of universal nuclease (Pierce) per ml of lysis buffer was added immediately prior to addition to the wells. Following incubation, samples were centrifuged at 17,000 × *g* at 4°C for 15 min to remove any insoluble material. Samples were quantitated using the EZQ protein quantitation kit (Pierce). Fifty micrograms of each sample was run in a 4% to 20% gradient SDS-PAGE gel (Bio-Rad) and transferred to a polyvinylidene difluoride (PVDF) 0.45-μm-pore-size membrane for 1 h at 300 mA. The membrane was probed using goat anti-MOMP (Meridian) and rabbit anti-HctB (generously provided by T. Hackstadt, NIH) primary antibodies followed by staining with donkey anti-goat 680 and donkey anti-rabbit 800 (LI-COR) secondary antibodies. The membrane was imaged on an Azure c600 imaging system. The channels were gray scaled and equally contrast corrected, and the resulting images were used for integrated density measurement with FIJI software (87). To assess relative HctB levels, the HctB integrated density of each sample was normalized to its respective MOMP integrated density to avoid bias due to lower overall organism numbers. The ratios were then used to compare induced versus uninduced relative HctB levels. These experiments were performed three times for a total of three biological replicates.

### Transmission electron microscopy assessment of the effect of overexpression of mutant Clp isoforms.

Samples were infected and induced as previously discussed (see above). At 48 hpi, samples were fixed using 2% glutaraldehyde, 2% formaldehyde in 0.1 M Sorensen’s phosphate buffer, pH 7.2. Samples were then stained postfixation in 1% osmium tetroxide in water for 1 h. Samples were dehydrated in an ethanol series (50%, 70%, 90%, 95%, and three changes of 100% ethanol), all steps 15 min each, and then soaked in propylene oxide (100%, 3 changes for 15 min each). Samples were left overnight in a fume hood in a 1:1 mixture of propylene oxide and Embed 812. The following day, the samples were placed in molds with fresh Embed 812 and polymerized overnight in an oven set at 65°C. Blocks were thin sectioned 90-nm thick on a Leica UC6 Ultramicrotome using a Diatome diamond knife. Sections were placed on uncoated 200 mesh copper grids and stained with 2% uranyl acetate and Reynold’s lead citrate. Sections were examined on a FEI Tecnai G2 transmission electron microscope (TEM) operated at 80 kV.

### Confirmation of *clpP2X* and *incA* knockdown.

Briefly, two wells of a six-well plate per condition were infected with pBOMBLCRia(*clpP2X*)::L2 transformed C. trachomatis L2 at a multiplicity of infection (MOI) of 0.8. At either 4 or 10 hpi, samples were or were not induced with 10 nM aTc. At each designated time point, total RNA was collected using TRIzol reagent (Invitrogen) and was extracted with chloroform as described previously (17, 83, 88–90). The aqueous layer was precipitated using isopropanol, as per the manufacturer’s instructions. Samples were DNase treated using the TURBO DNA-free kit (Ambion), and 1 μg of the resulting RNA was reverse transcribed using SuperScript III reverse transcriptase (Invitrogen). Equal volumes of cDNA were loaded for each qPCR. To extract genomic DNA, one well per condition was harvested and processed using the DNeasy blood and tissue kit (Qiagen) according to the manufacturer’s instructions as noted above. Samples were diluted to 5 ng/μl, and 5 μl of the resulting dilution was used per qPCR. cDNA and gDNA samples were quantified using 25-μl reaction mixtures with 2× SYBR PowerUP green master mix (Invitrogen) analyzed on a QuantStudio 3 (Applied Biosystems) thermal cycler using the standard cycling conditions. A standard curve using purified wild-type C. trachomatis L2 genomic DNA was generated for sample quantification. Data are displayed as the ratio of cDNA to gDNA normalized to the 10-h uninduced sample. For *incA* knockdown, HEp-2 cells were infected with the pBOMBLCRia(*incA*)::L2 transformant, induced with 10 nM aTc as described above, and fixed at 24 hpi with methanol. Cells were labeled with primary guinea pig anti-major outer membrane protein (MOMP; kind gift of E. Rucks, UNMC), rabbit anti-Sa_dCas9 (Abcam, Cambridge, MA), and sheep anti-IncA (E. Rucks) antibodies and DAPI. Appropriate donkey secondary antibodies were used (Invitrogen). Coverslips were mounted on glass slides using ProLong Glass Antifade mounting medium (Thermo Fisher). Images were acquired on a Zeiss Axio ImagerZ.2 equipped with Apotome.2 optical sectioning hardware and X-Cite Series 120PC illumination lamp.

### Determination of the effect of *clpP2X* knockdown on C. trachomatis.

Twenty-four-well plates of HEp-2 cells were infected at an MOI of 0.8 with either pBOMBLCRia(*clpP2X*)::L2 or pBOMBLCRia(*incA*)::L2 transformed into C. trachomatis L2. Samples were induced or not at 4 hpi and were harvested and fixed, and the titer was determined as previously described. To monitor the expression of dCas9 and, if present, the complemented gene of interest, immunofluorescence samples on glass coverslips were fixed and permeabilized using ice-cold 90% methanol at 24 or 48 hpi. Each sample was then stained using goat anti-MOMP (Meridian BioScience), rabbit anti-Sa_dCas9, and mouse anti-FLAG (Sigma-Aldrich) primary antibodies. Samples were then stained with the appropriate donkey secondary antibodies and were imaged as discussed above. Each titration was fixed using 4% formaldehyde and 0.025% glutaraldehyde to preserve GFP fluorescence. IFU counts of GFP-positive inclusions are displayed as a percentage of the uninduced sample at the designated time point. Plasmid retention for each condition is displayed as the percent GFP positive to total number of inclusions for each condition.

### GFP_SsrA stability assay.

Chlamydial transformants harboring either pBOMBmC-GFP(VAA) or pBOMBmC-GFP(VDD) plasmids were used to infect a fresh monolayer of McCoy mouse fibroblasts on coverslips in a 24-well plate. Samples were induced or not at 8 hpi. At 16 hpi, all media were replaced with fresh DMEM with or without the designated compound. GFP expression was either continued through 20 hpi or was removed from 16 to 20 hpi for pulse-chase samples. At 20 hpi, all samples were formaldehyde-glutaraldehyde fixed as previously discussed. Coverslips were then mounted and imaged as previously discussed using a 40× lens objective. GFP and mCherry integrated densities were measured for approximately 60 fields of view on two separate coverslips per condition. These experiments were repeated for two biological replicates. To determine the ratio of GFP to mCherry, GFP integrated density measurements were divided by those for mCherry.

### Effect of ClpX-targeting compounds on chlamydial growth and host cell viability.

Stocks of ClpX-specific inhibitor 334 and its derivative, 365, were synthesized as previously reported (44), resuspended at 25 mg/ml in DMSO, and frozen at −20°C. Methods for the synthesis, purification, and analysis of these compounds are available upon request. A dose curve for treatment was assessed to determine an inhibitory concentration of the compounds on C. trachomatis, and 25 μg/ml was chosen (data not shown). For the 24- and 48-h samples, 500 μl of DMEM containing 25 μg/ml of the compounds was added at 8 hpi, and samples were harvested at the designated time point. For the time of infection samples, compounds were added at 15 min postinfection and removed at 8 hpi. For the reactivation samples, DMEM containing the respective compound was added at 8 hpi, washed out three times with HBSS at 24 hpi, and then replaced with DMEM only for 24 additional hours prior to harvest. To determine the effect on preformed EBs, compound was added at 24 hpi, and samples were harvested at 48 hpi. To harvest, three wells of a 24-well plate were scraped into 2SP, vortexed with 3-mm glass beads, and frozen at −80°C. Samples were titrated onto a fresh monolayer of HEp-2 cells with no treatment for enumeration. For assessment of cell viability, HEp-2 cells were plate in a 96-well dark-wall plate and were treated as described for IFU experiments. All wells were then mixed with PrestoBlue HS reagent (Invitrogen), incubated for 10 min, and measured for fluorescence using a Tecan plate reader. Values are given as percentages of the untreated sample.
