# Supplementary figures and images for: The ClpX and ClpP2 Orthologs of Chlamydia trachomatis Perform Discrete and Essential Functions in Organism Growth and Development
Source: mBio. 2020 Sep 1;11(5):e02016-20. doi: 10.1128/mBio.02016-20 (PMC7468207; doi:10.1128/mBio.02016-20)

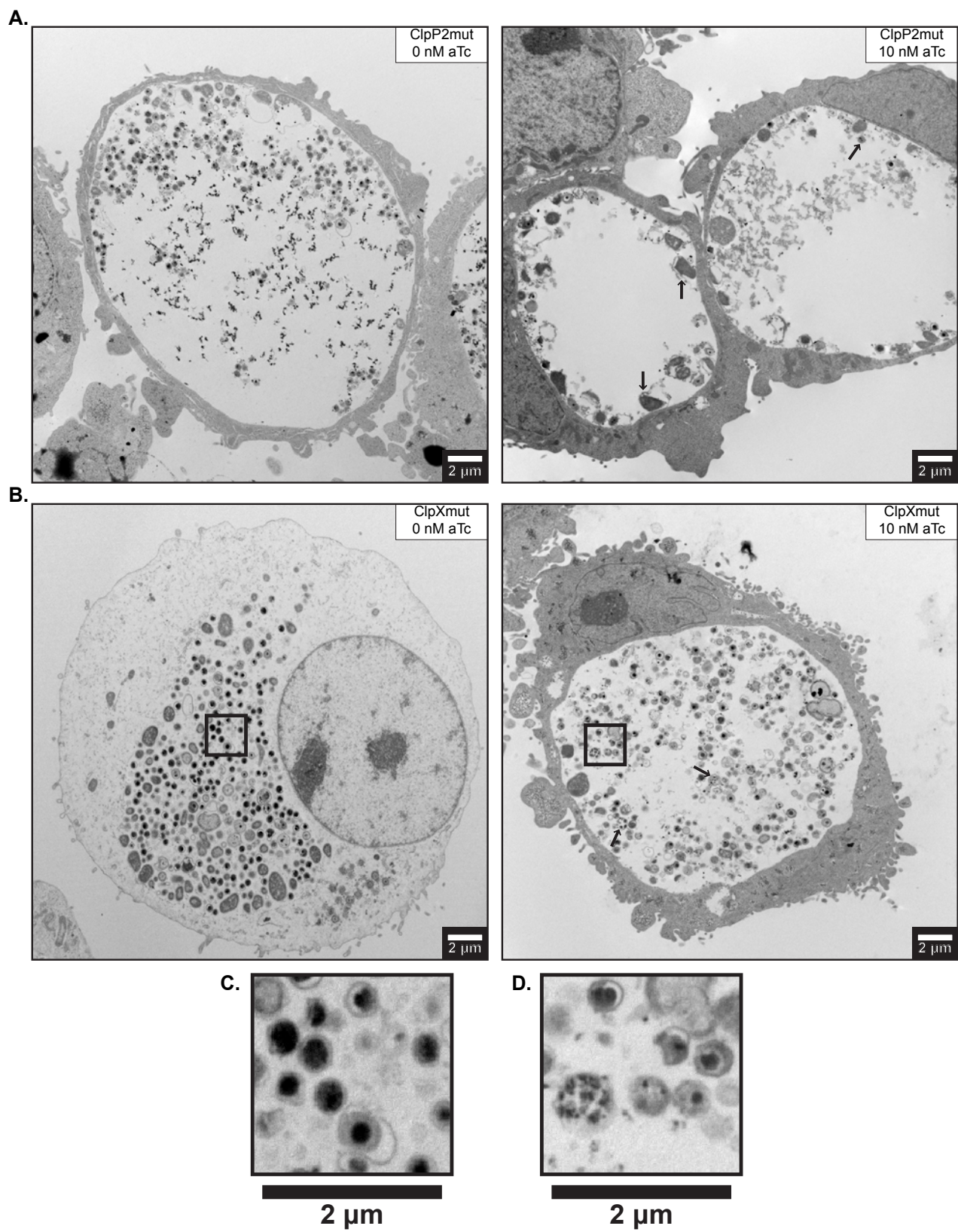

**Fig. S1**

Supplement: FIG S1 [file mBio.02016-20-sf001.pdf]

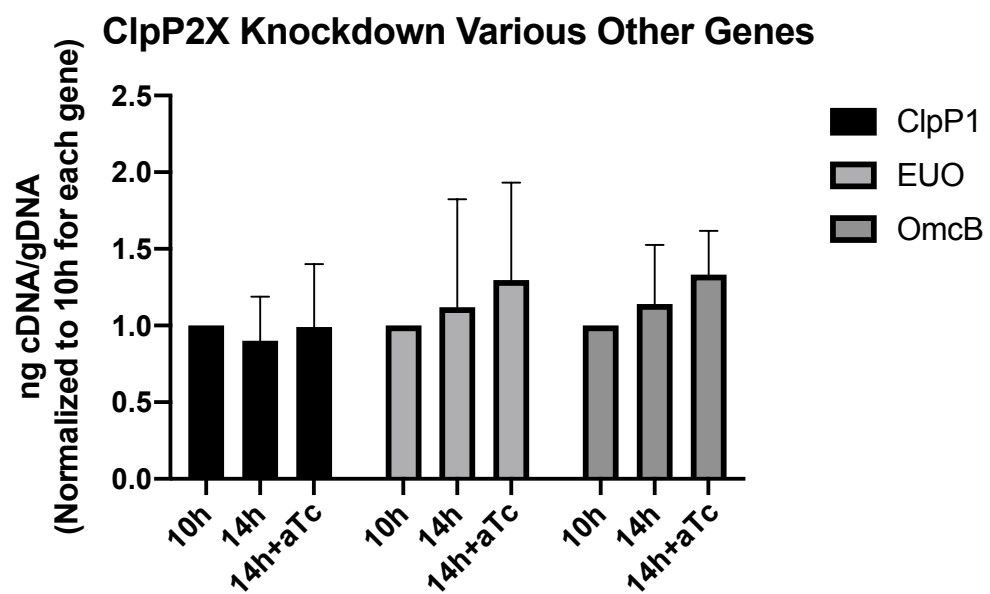

**Fig. S2**

Supplement: FIG S2 [file mBio.02016-20-sf002.pdf]

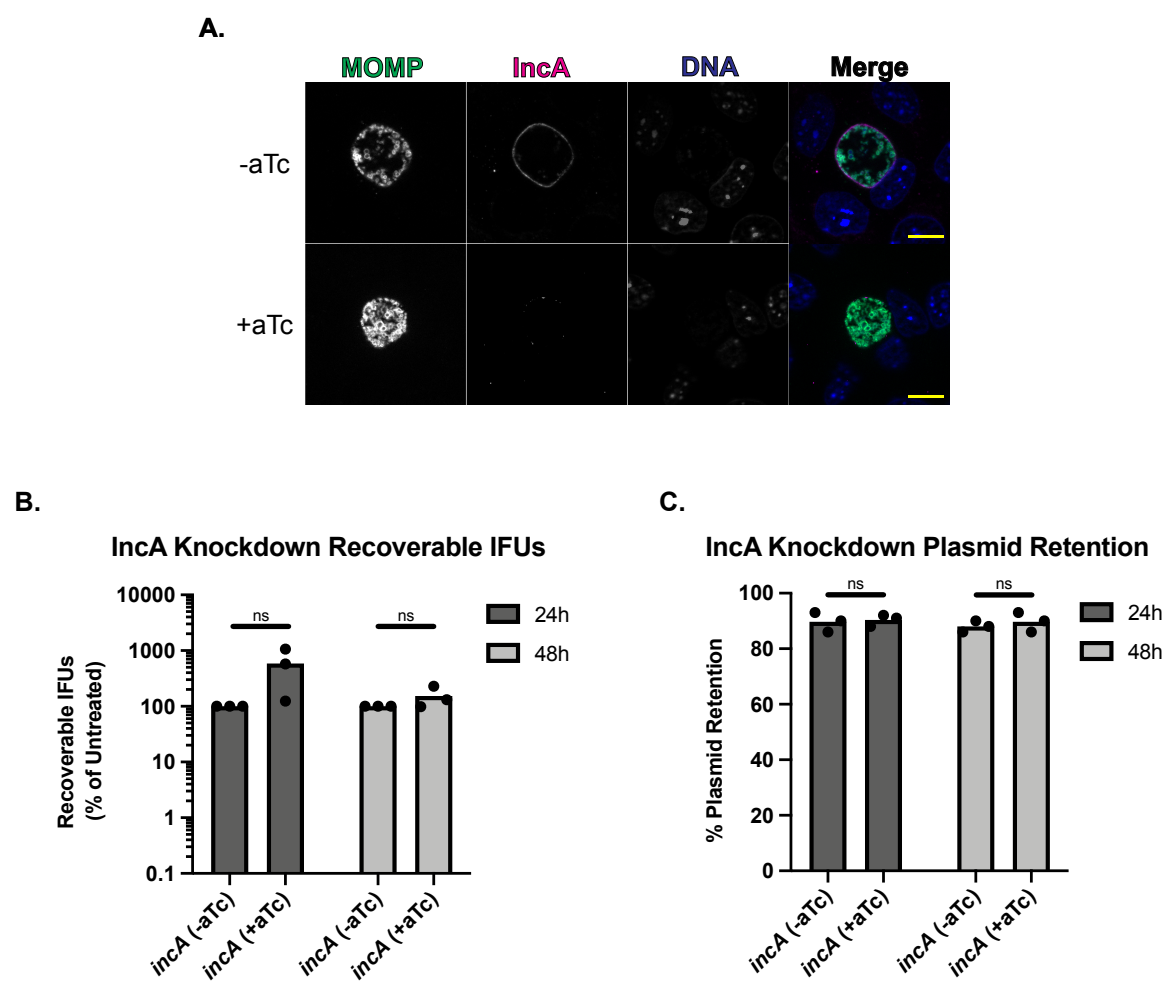

**Fig. S3**

Supplement: FIG S3 [file mBio.02016-20-sf003.pdf]

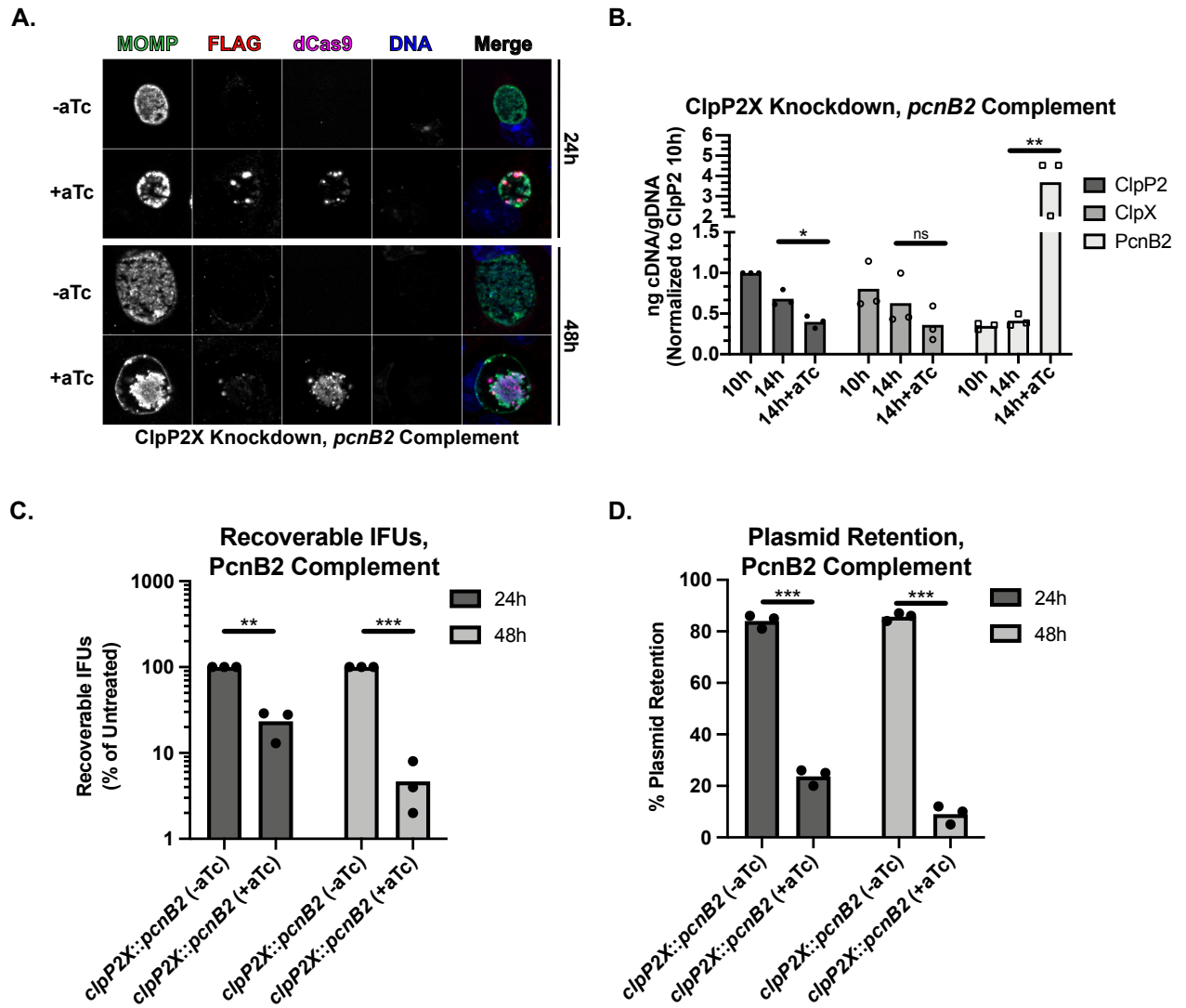

Fig. S4

Supplement: FIG S4 [file mBio.02016-20-sf004.pdf]

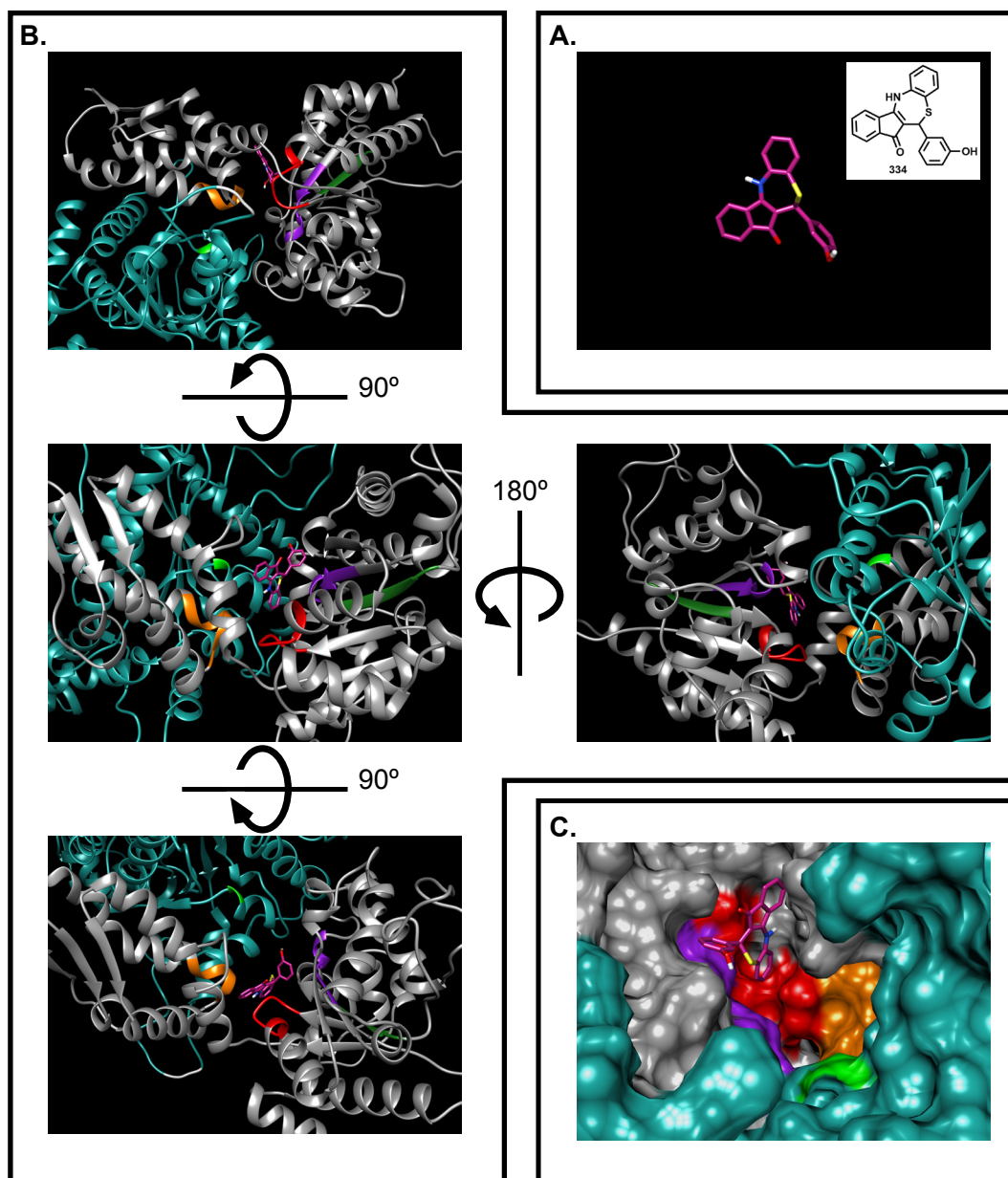

Fig. S5

Supplement: FIG S5 [file mBio.02016-20-sf005.pdf]

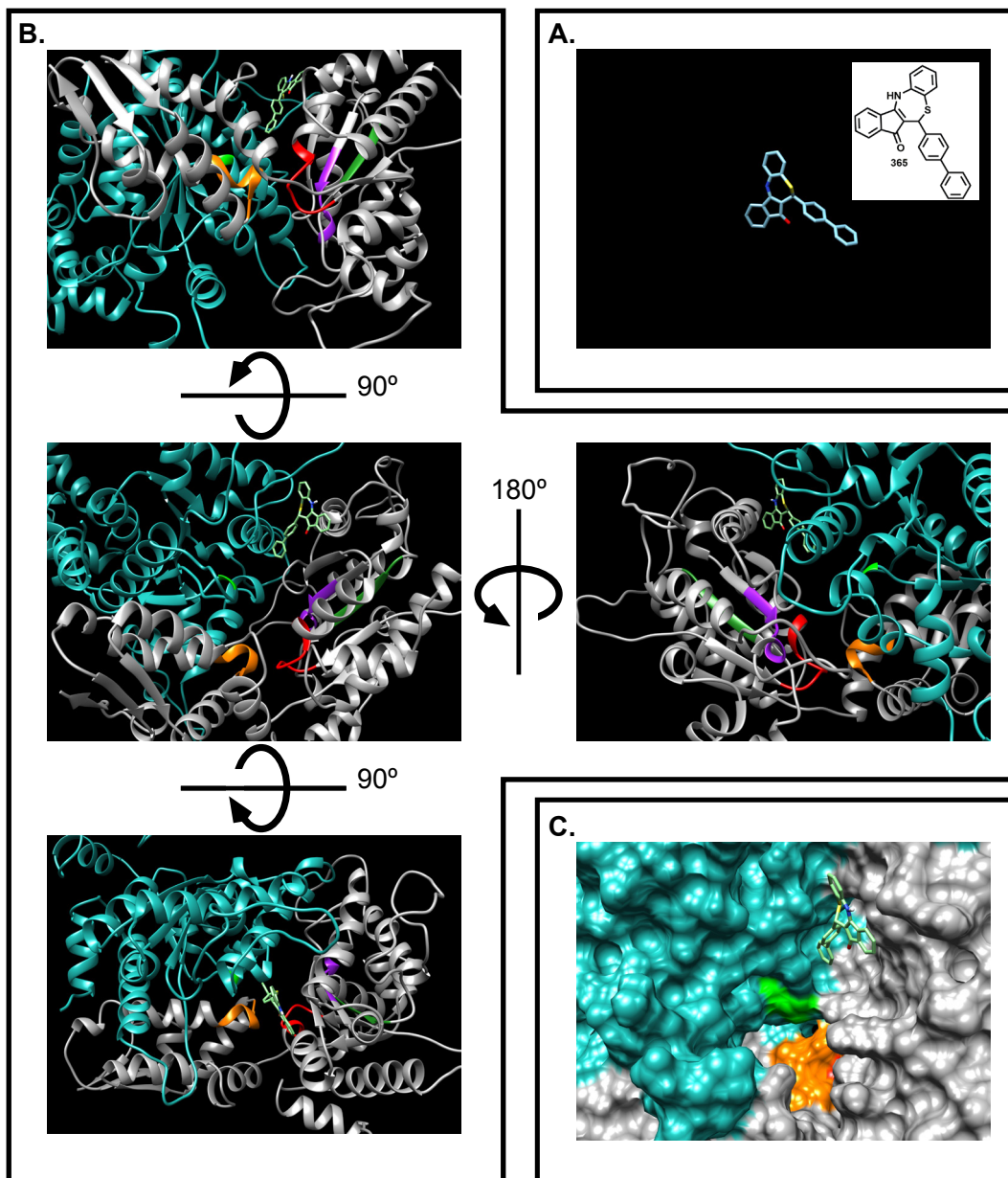

Fig. S6

Supplement: FIG S6 [file mBio.02016-20-sf006.pdf]

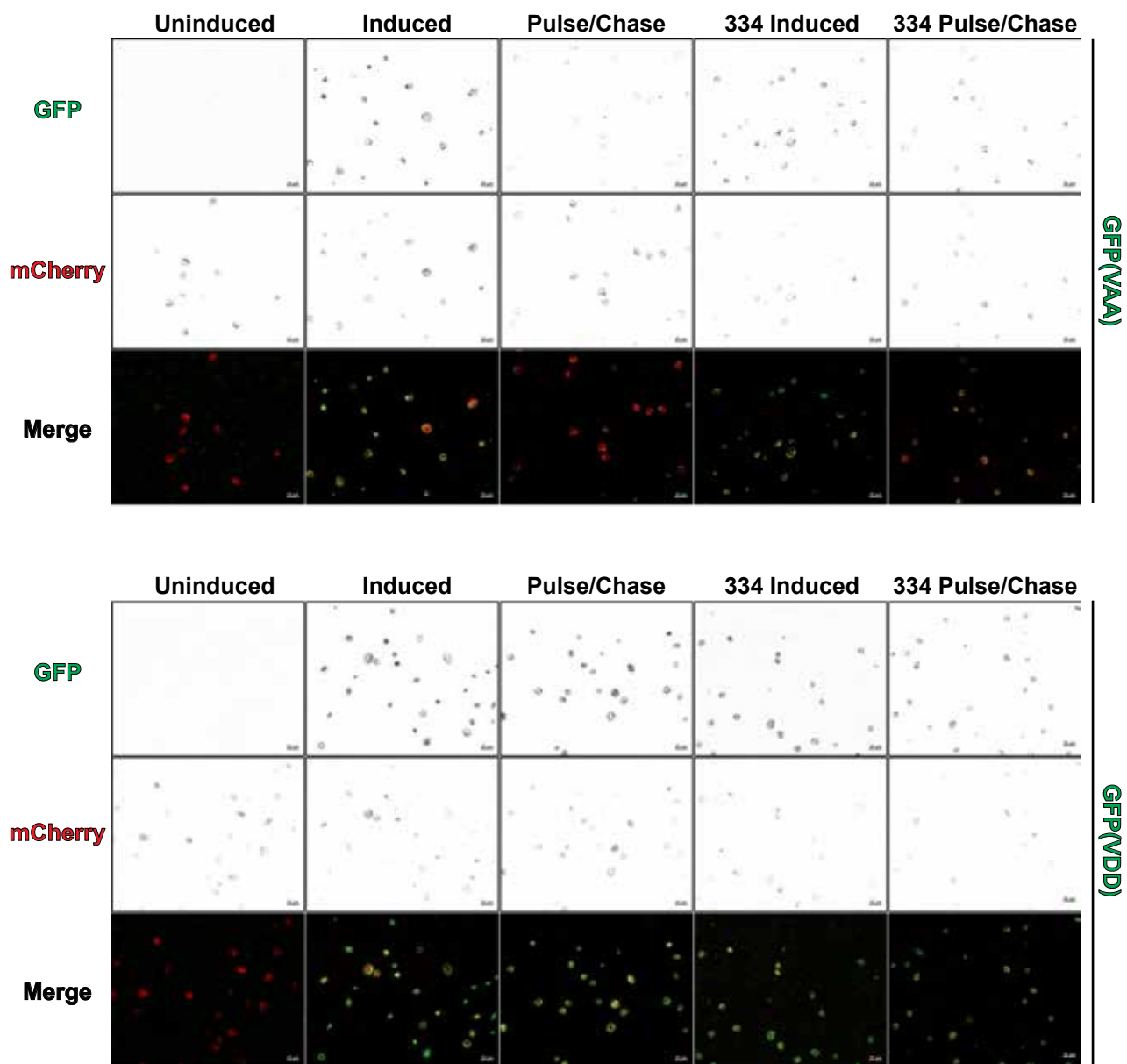

Fig. S7

Supplement: FIG S7 [file mBio.02016-20-sf007.pdf]

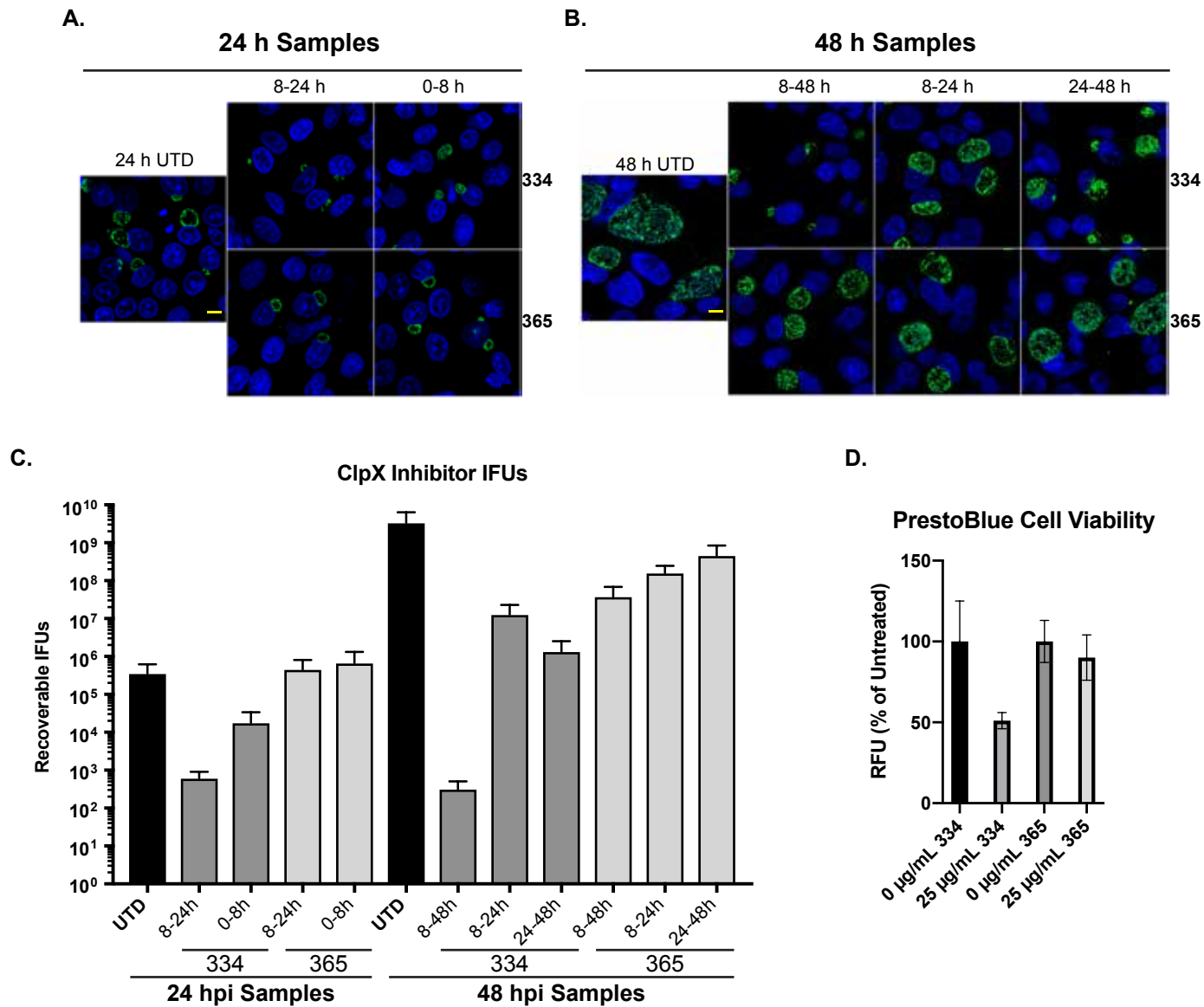

**Fig. S8**

Supplement: FIG S8 [file mBio.02016-20-sf008.pdf]

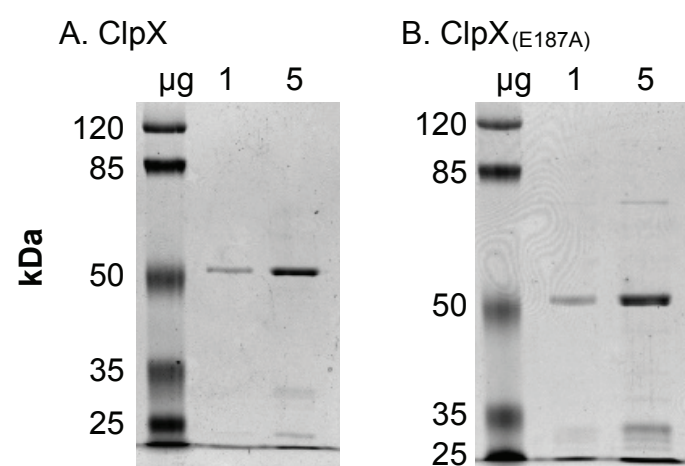

Fig. S9

Supplement: FIG S9 [file mBio.02016-20-sf009.pdf]
